# The First Dimeric Derivatives of the Glycopeptide Antibiotic Teicoplanin

**DOI:** 10.3390/ph15010077

**Published:** 2022-01-08

**Authors:** Ilona Bereczki, Zsolt Szűcs, Gyula Batta, Tamás Milán Nagy, Eszter Ostorházi, Katalin E. Kövér, Anikó Borbás, Pál Herczegh

**Affiliations:** 1 Department of Pharmaceutical Chemistry, University of Debrecen, Egyetem tér 1, H-4032 Debrecen, Hungary; bereczki.ilona@pharm.unideb.hu (I.B.); zsolty123@gmail.com (Z.S.); 2 Department of Organic Chemistry, University of Debrecen, H-4032 Debrecen, Hungary; batta@unideb.hu; 3 Department of Inorganic and Analytical Chemistry, University of Debrecen, H-4032 Debrecen, Hungary; tamasmilan.nagy@science.unideb.hu (T.M.N.); kover@science.unideb.hu (K.E.K.); 4 MTA-DE Molecular Recognition and Interaction Research Group, University of Debrecen, H-4032 Debrecen, Hungary; 5 Department of Medical Microbiology, Semmelweis University, Nagyvárad tér 4, H-1089 Budapest, Hungary; ostorhazi.eszter@med.semmelweis-univ.hu

**Keywords:** teicoplanin, pseudoaglycone, synthesis, dimer, Co(III) complex, DOSY NMR, antibacterial activity

## Abstract

Various dimeric derivatives of the glycopeptide antibiotic teicoplanin were prepared with the aim of increasing the activity of the parent compound against glycopeptide-resistant bacteria, primarily vancomycin-resistant enterococci. Starting from teicoplanin, four covalent dimers were prepared in two orientations, using an α,ω-bis-isothiocyanate linker. Formation of a dimeric cobalt coordination complex of an *N*-terminal L-histidyl derivative of teicoplanin pseudoaglycone has been detected and its antibacterial activity evaluated. The Co(III)-induced dimerization of the histidyl derivative was demonstrated by DOSY experiments. Both the covalent and the complex dimeric derivatives showed high activity against VanA teicoplanin-resistant enterococci, but their activity against other tested bacterial strains did not exceed that of the monomeric compounds.

## 1. Introduction

Glycopeptide antibiotics vancomycin, teicoplanin, oritavancin, dalbavancin and telavancin have strong therapeutic potential in cases of severe infections caused by Gram-positive bacteria, such as multidrug-resistant *Staphylococcus aureus,* or enterococci [1,2,3,4,5,6]. The antibacterial activity of vancomycin and teicoplanin is based mainly on their binding to the D-alanyl-D-alanine terminal part of the pentapeptide of the lipid II unit of the bacterial peptidoglycan, thus inhibiting the transpeptidation and transglycosylation phase of peptidoglycan biosynthesis [6]. It has long been known that most of the glycopeptide antibiotics, e.g., ristocetin [7] and eremomycin [8], form non-covalent, reversible dimers in aqueous solutions, and strong dimerization is considered to be beneficial for antibacterial effects [9]. Multivalency or chelate effects support this view, since after one site is bound, the second binding occurs effectively in an intramolecular way. Induction of ligand-induced formation of higher glycopeptide oligomers was demonstrated both in crystal state [10] and in aqueous solution [11,12,13]. Studies on vancomycin revealed that the non-covalent dimerization aptitude of the vancomycin molecule can be an important factor of its antibacterial activity, as the dimerization promotes its affinity/co-operativity to the ligands, i.e., D-alanyl-D-alanine part of the bacterial peptidoglycan [10,14,15,16,17,18]. Motivated by these observations, numerous covalent dimers of vancomycin and eremomycin have been synthesized in order to improve the antibacterial activity of the parent molecule [19,20,21,22,23,24,25,26,27,28,29]. Indeed, some of these covalent dimers showed enhanced activity against resistant bacteria, such as vancomycin-resistant enterococci type VanA.

**Scheme 1:**
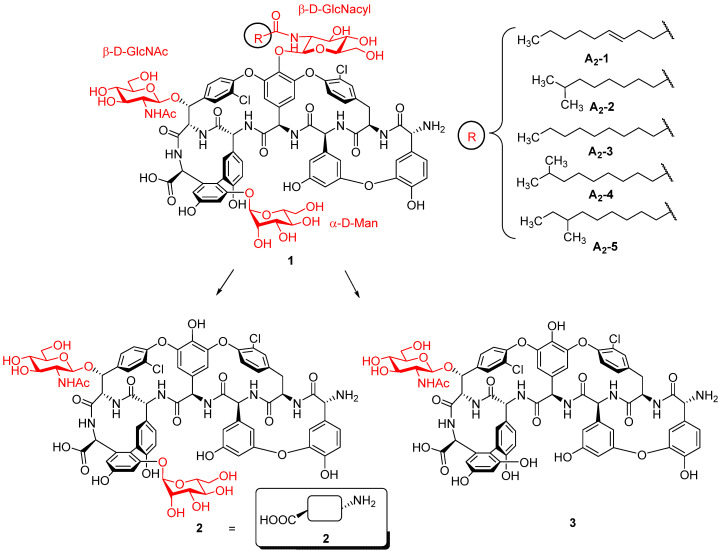
Conversion of teicoplanin **1** into teicoplanin pseudoaglycones **2** and **3**. The sugar units are highlighted in red.

Teicoplanin (**1**) is not able to form non-covalent dimers because of its apolar tag, and its covalent dimers are also unknown. Teicoplanin used in the therapy is a mixture of five closely related derivatives, mainly consisting of the A_2_ component, differing from each other in the *N*-acyl substituent of one of the D-glucosamine moieties (Scheme 1). The separation of these components is difficult; therefore, synthetic modifications of the teicoplanin mixture also lead to complicated mixtures. Removal of the variously *N*-acylated apolar D-glucosamine substituent from **1** by partial hydrolysis results in a single aglycone derivative **2** bearing two sugar units [30]. In recent years, our group has been dealing with systematic synthetic modifications of teicoplanin pseudoaglycone **3**, which can be obtained from teicoplanin mixtures as a single compound with removal of mannose and variously *N*-acylated D-glucosamine moieties [31]. In the framework of these synthetic studies, we introduced lipophilic substituents in compounds **2** and **3**, obtaining derivatives that formed nanoaggregates in solution and were highly active against resistant staphylococci and enterococci [31,32,33]. Notably, dimers of neither the pseudoaglycone **2** with two sugar units nor the pseudoaglycone **3** bearing a single GlucNAc residue have been prepared yet. Therefore, we decided to synthesize some of these derivatives in order to study the influence of dimerization on the antibacterial activity in the teicoplanin series. In this work, we report on the synthesis of covalent dimers of **2** together with a study on a cobalt complex dimer obtained from a derivative of **3**.

## 2. Results and Discussion

**Scheme 2:**
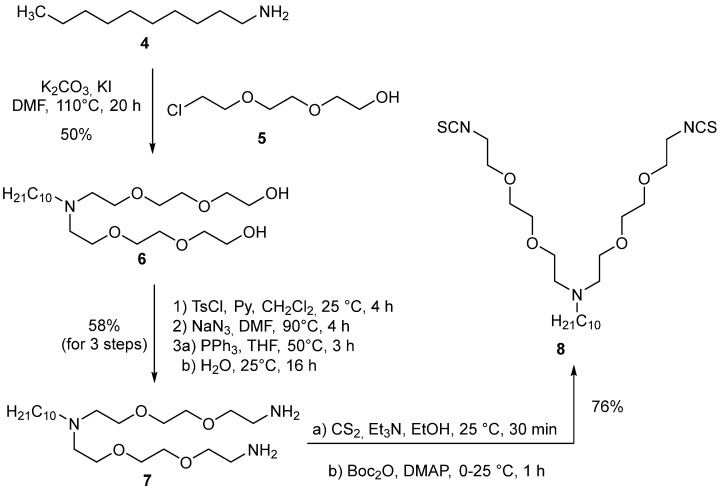
Syntheses of the α,ω-bis-isothiocyanate linker **8**.

**Scheme 3:**
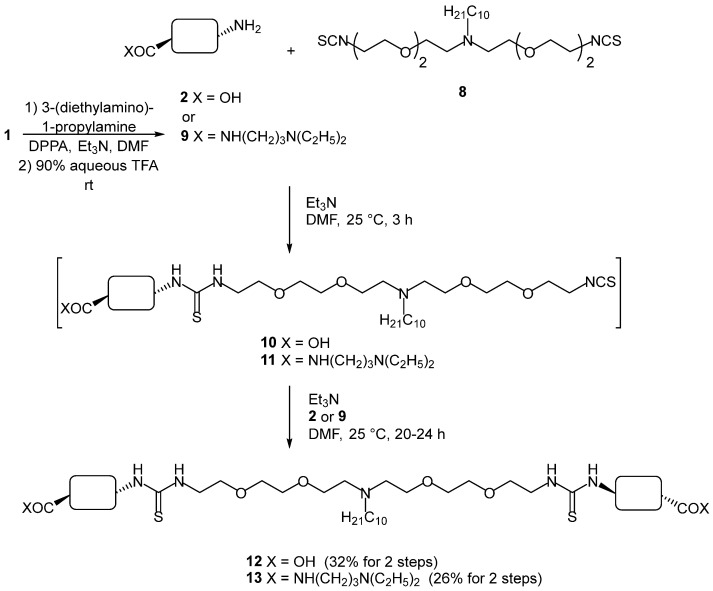
Synthesis of covalent dimers of teicoplanin pseudoaglycone **2** with *N*-*N* orientation.

**Scheme 4:**
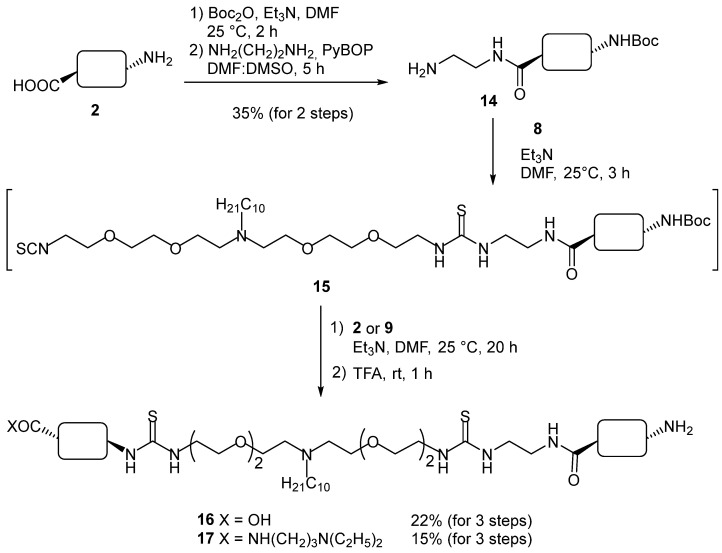
Synthesis of *N-C* dimers from teicoplanin pseudoaglycone **2**.

For covalent dimer formation, simple linker reagent **8** was prepared with two reactive isothiocyanate groups and a lipophilic *n*-decylamino substituent to give an amphiphilic character to the dimers. Reacting *n*-decylamine **4** with a chloro derivative of triethylene glycol (**5**), tertiary amine-diol **6** was obtained. The hydroxyl groups of **6** were transformed to primary amino groups in a sequence of tosylation, nucleophilic substitution with azide and Staudinger reduction to give **7**. Transformation [34] of the amino groups in compound **7** resulted in linker reagent **8** (Scheme 2). Linker **8** can tether two glycopeptides through thiourea bonds by reacting its two isothiocyanate functionalities with either the *N*-terminal amino group of compound **2** (Scheme 3) or an amino group attached to the *C*-terminus of **2** (Scheme 4).

Using this diisothiocyanate **8**, covalent dimers of **2** and its diethylaminopropyl amide **9** were prepared with an *N,N*-terminal orientation. For this reason, the carboxy-terminus of teicoplanin was converted into diethylaminopropyl amide, similar to the same structural element of the very potent antibacterial dalbavancin, and the *N*-acyl-glucosamine moiety with different side chains was removed hydrolytically to yield **9** [35]. Additionally, **2** or **9** was reacted with excess **8**, and after workup, the obtained monoisothiocyanates **10** and **11** were reacted with an equivalent of **2** or **9** to give the dimers **12** and **13** (Scheme 3). In preliminary attempts, the linking of the two identical monomers was also tried using exactly half equivalent of the linker. Unfortunately, this approach led to sluggish conversion into dimers and reaction mixtures with significantly more components compared to the two-step method with the excess linker, making the already difficult purification even more challenging. The new two-step method gave much better control over the course of the reaction. The major disadvantage is obviously the waste of the excess linker if not recovered by chromatography.

Two more dimers with *N,C* terminal orientation were obtained from pseudoaglycone **2** (Scheme 4). The amino group of **2** was protected in the form of *t*-butyl carbamate and subsequently the carboxylic moiety was converted to an aminoethyl amide to produce **14**. The primary amino group of **14** allowed its coupling to linker **8** through a *C*-terminal thiourea linkage to produce monoisothiocyanate derivative **15**. Compound **15** was then reacted with either **2** or its diethylamino propylamide derivative **9**, resulting in the two dimers **16** and **17** after hydrolytic removal of the Boc group.

**Scheme 5:**
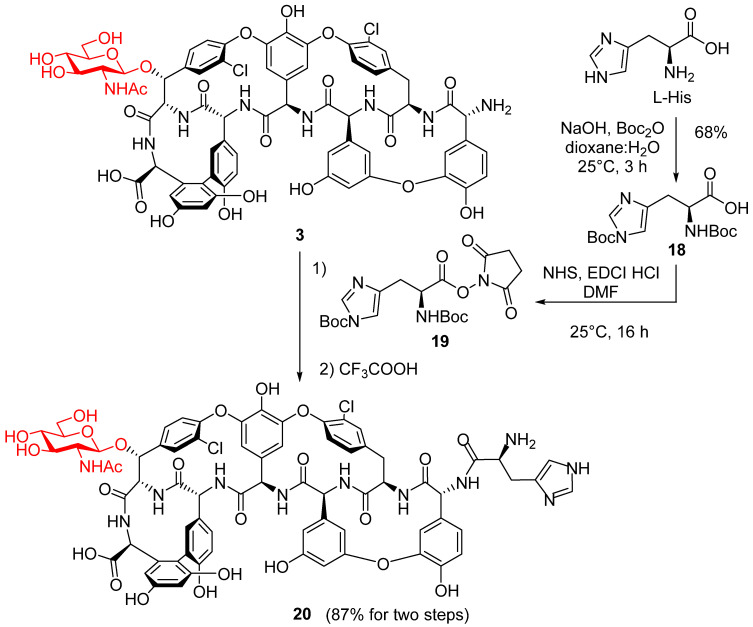
Synthesis of the histidyl conjugate of teicoplanin pseudoaglycone **3**.

**Figure 1:**
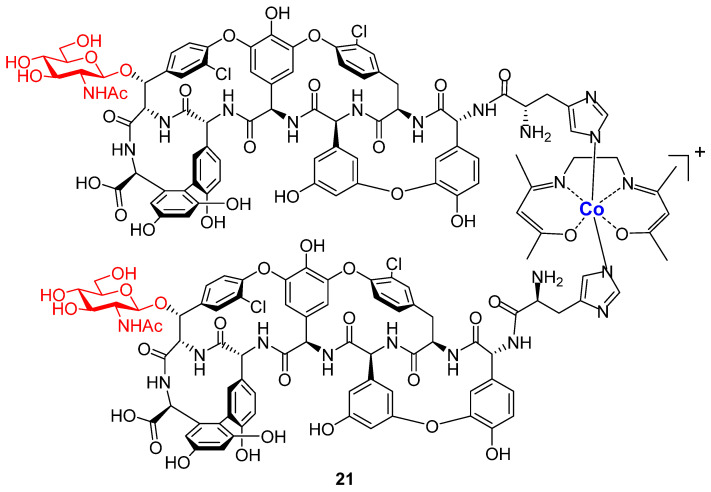
Structure of the Co(III) complex dimer obtained from **3**.

We also used another approach for the formation of the *N,N* dimer. Meade and coworkers studied the stability and interaction of Schiff base cobalt diammine coordination complexes bearing labile axial ligands with histidine [36,37]. These complexes contain an acetylacetonatoethylenediimine (acacen) [38] equatorial ligand stabilizing the Co^3+^ center. They observed a strong and selective binding of histidine or histidine parts of proteins to the complex. This reaction is based on a dissociative axial ligand exchange. We used this selective coordination complex formation to obtain a dimer from teicoplanin pseudoaglycone **3**. Since this compound does not contain a histidine moiety, we prepared a histidyl derivative of **3**. For this purpose, from the di-Boc derivative of histidine (**18**) [39], an *N-*hydroxysuccinimide ester (**19**) was obtained. With the use of the latter, the *N-*terminal amino group of **3** was acylated and the carbamate protecting groups of the intermediate were removed by trifluoroacetic acid treatment to give **20** (Scheme 5). This compound reacted with the Co^3+^ acacen Schiff base complex to produce dimeric derivative **21** (Figure 1).

**Figure 2:**
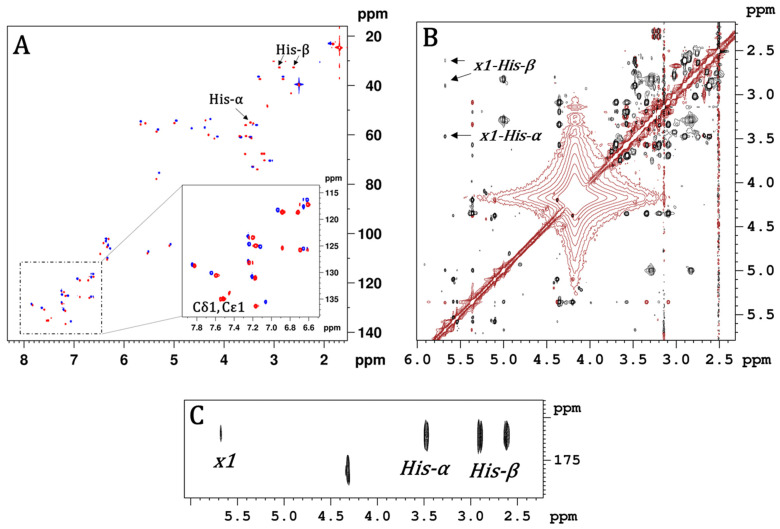
Excerpts of 2D homo- and heteronuclear correlation maps. (**A**) Overlay of the ^13^C-^1^H HSQC spectra of teicoplanin pseudoaglycone **3** (blue) and its histidyl conjugate **20** (red). Additional resonances arise from the histidyl group atoms (aromatic Cδ1, Cε1 and Hα, Hβ correlations are labeled). (**B**): Selected ^1^H-^1^H ROESY spectral region indicates the spatial proximity (<0.5 nm) of histidyl Hα and Hβ atoms to the Hα of the first residue (x1). (**C**): ^13^C-^1^H HMBC excerpt demonstrates correlation between the carbonyl atom and x1 proton as proof of connectivity between the glycopeptide chain and the histidyl group. Intraresidual correlations of histidyl carbonyl with Hα and Hβ are also labeled. The resonance assignments for the identified atoms are as follows: 54.74 ppm (Cα), 32.38 ppm (Cβ), 135.15 ppm (135.00 ppm (Cδ1), 135.08 ppm (Cε1), 174.41 ppm (CO), 3.47 ppm (Hα), 2.89 ppm (Hβ1), 2.61 ppm (Hβ2), 7.55 ppm (Hδ1), and 7.52 ppm (Hε1).

**Figure 3:**
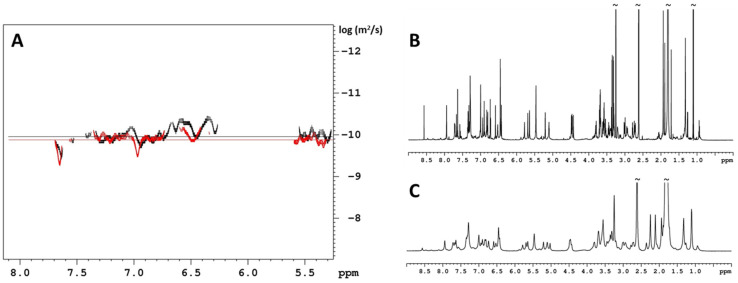
500 MHz ^1^H DOSY and ^1^H NMR spectra. (**A**) Overlay of DOSY plots of **20** (red) and its Co-complex **21** (black). The slightly slower diffusion of **21** supports the formation of dimeric species. (**B**) and (**C**) ^1^H NMR spectra of **20** and **21**, respectively. The line-broadening observed in the ^1^H NMR spectrum of **20** (shown in **C**) upon addition of Schiff base cobalt diammine indicates a monomer-dimer equilibrium in the intermediate regime on the ^1^H NMR timescale.

The structure of the histidyl derivative (**20**) and the formation of a dimer (**21**) induced by the cobalt compound was verified by NMR spectroscopy in detail (Figure 2). First, the ^1^H and ^13^C resonances of the newly introduced histidyl group were unambiguously identified on the basis of one- (1D) and two-dimensional (2D) correlation experiments (Figure 2A). The linkage between the pseudoaglycone and histidyl group was confirmed by the through-space ROESY connectivities observed between x1(H)-His(Hα) and x1(H)-His(Hβ) protons (Figure 2B) and also by the multiple-bond heteronuclear (^1^H/^13^C) correlations detected between the carbonyl (CO) carbon and the x1(H)/His(Hα)/His(Hβ) protons, respectively (Figure 2C). The formation of dimeric species upon addition of the Co-complex was monitored by Diffusion Ordered Spectroscopy (DOSY) and allowed the measurement of the translational diffusion coefficients of **20** and **21** as 1.79 × 10^−10^ m^2^/s and 1.52× 10^−10^ m^2^/s, respectively (Figure 3A). The slightly slower diffusion of the Co-complex suggests the formation of a dimer being in equilibrium with the monomeric form. In addition, the significant broadening of ^1^H resonances in Figure 3C (compared to Figure 3B) indicates that the dynamics of this monomer–dimer equilibrium fall in the intermediate regime on the ^1^H NMR timescale.

The antibacterial activity of the dimeric compounds was evaluated on a panel of Gram-positive bacteria (Table 1). As can be seen, *N,N*-terminal dimer **12** displayed very diminished activity in comparison to that of the teicoplanin mixture **1**. The formation of a basic amide in **13**, similar to dalbavancin, was beneficial to the antibacterial activity, which was still a bit smaller than that of the parent teicoplanin **1**. Moderate antibacterial activities were obtained for the two *N,C* dimers **16** and **17**. However, it is remarkable that aglycone **2** and all dimers **12**, **13**, **16** and **17** displayed relatively high activity against VanA teicoplanin-resistant enterococci. Interestingly, the dimeric derivatives of teicoplanin in general displayed somewhat lower activity against enterococci with the VanB resistance gene and also against nonresistant enterococci ATCC 29212. Since the mechanism of action of glycopeptide antibiotics is based on the inhibition of cell wall peptidoglycan, this phenomenon can be explained by the different structures of the cell walls of these enterococci strains. Similarly, a low MIC value (4 μg/mL) was detected for compound **20**, a histidyl derivative of **3**. Unfortunately, cobalt complex dimerization of the same derivative did not improve the antibacterial activity of the monomer (see the activity of dimer **21** in Table 1). In this case, it has to be noted that complex formation is an equilibrium phenomenon and, consequently, the activity measured for **21** is attributable to the equilibrium mixture of **20** and **21**.

## 3. Materials and Methods

### 3.1. General Information

*n*-Decylamine (**4**), 2-[2-(2-chloroetoxy)ethohy]etanol (**5**), 3-(diethylamino)-1-propylamine and L-histidine are commercially available. *N*,*N*′-di-Boc-L-histidine (**18**) was prepared by the literature procedure [39].

The antibacterial evaluations were carried out as described in our previous publication [40]. TLC was performed on Kieselgel 60 F_254_ (Merck) with detection either by immersion in ammonium molybdate–sulfuric acid solution followed by heating or by using Pauly’s reagent for detection. Flash column chromatography was performed using Silica gel 60 (Merck 0.040–0.063 mm).

NMR experiments for structure elucidation (^13^C-^1^H HSQC, HMBC (using 80 ms for ^n^*J*_CH_ evolution), HSQC-TOCSY (with 80 ms TOCSY mixing time) and ^1^H-^1^H ROESY (with 60 ms mixing time) were carried out on a Bruker Avance Neo 700 MHz spectrometer equipped with a Prodigy TCI cryoprobe. The temperature was set to 298 K, and chemical shifts were referenced to the solvent signals. A sample of teicoplanin pseudoaglycone **3** (4.7 mg) was dissolved in 550 μL DMSO-d_6_, while the histidyl derivative **20** (10 mg) was dissolved in a mixture of 400 μL DMSO-d_6_ and 150 μL MeOD.

Diffusion Ordered SpectroscopY (DOSY) measurements were carried out at 298 K using a 500 MHz Bruker Avance II spectrometer equipped with TXI (^1^H/^13^C/^15^N triple resonance) probe. Diffusion coefficients were determined with the stimulated spin echo LED (Longitudinal-Eddy-current Delay) sequence (ledbpgp2s) of the Bruker pulse sequence library. The length of the diffusion delay (50 ms) and gradient pulses (5 ms) were optimized by short, one-dimensional (1D) experiments. The gradient strength was increased in 32 increments, linearly varying between 5% and 95% of its maximum value. The maximum gradient strength of the probe is 57.7 G/cm. The number of scans acquired for each increment was 16. Histidyl derivative teicoplanin pseudoaglycone (2.07 mg) was dissolved in 25 μL DMSO-d_6_ and 475 μL D_2_O mixture for measurement. Then, 4 μL of Co-complex from a stock solution (5.11 mg/100 μL) was added to the sample and the measurement was repeated with the same experimental setup. The dosy module of Topspin 2.1. was used for data processing and generation of 2D DOSY plots.

The ^1^H NMR (360 and 400 MHz), ^13^C NMR (90 and 100 MHz) and 2D NMR spectra were recorded with Bruker DRX-360 and Bruker DRX-400 spectrometers at 25 °C. Chemical shifts are referenced to Me_4_Si (0.00 ppm for ^1^H) and to the solvent residual signals. The spectra were evaluated using MestReNova and TopSpin software.

MALDI-TOF MS measurements were carried out with a Bruker Autoflex Speed mass spectrometer equipped with a time-of-flight (TOF) mass analyzer. In all cases, 19 kV (ion source voltage 1) and 16.65 kV (ion source voltage 2) were used. For reflectron mode, 21 kV and 9.55 kV were applied as reflector voltage 1 and reflector voltage 2, respectively. A solid-phase laser (355 nm, ≥100 μJ/pulse) operating at 500 Hz was applied to produce laser desorption and 3000 shots were summed. 2,5-Dihydroxybenzoic acid (DHB) was used as the matrix and F_3_CCOONa as the cationizing agent in DMF. A MicroTOF-Q type Qq-TOF MS instrument (Bruker Daltonik, Bremen, Germany) was used for the ESI-MS measurements. The instrument was equipped with an electrospray ion source where the spray voltage was 4 kV. N_2_ was utilized as the drying gas. The drying temperature was 180 °C and the flow rate was 4.0 L/min. The mass spectra were recorded by means of a digitizer at a sampling rate of 2 GHz. The spectra were evaluated with DataAnalysis 3.4 software from Bruker (Bremen, Germany).

For analytical RP-HPLC, a Waters 2695 Separations Module (Waters Corp., Milford, ME, USA) was used. The separations were carried out on a VDSpher PUR 100 C18-M-SE, 5 μm, 150 × 4.6 mm column (Batch# VD173001) at an injection volume of 10 μL, using a flow rate of 1.0 mL/min with a Waters 2996 DAD set at 254 nm and a Bruker MicroTOF-Q type Qq-TOF MS instrument (Bruker Daltonik, Bremen, Germany) as detectors. The following system was used for the elutions: Solvent A: Water:MeCN 9:1 + 0.0025% *v*/*v* TFA, Solvent B: MeCN. Gradient: 20% B from 0 to 20 min, from 20% B to 80% B from 20–40 min, 80% B from 40 to 50 min, from 80% B to 20% B from 50 to 51 min. Solvent A: Water: MeCN 9: 1 + 0.0025% *v*/*v* TFA, Solvent B: MeCN. The MicroTOF-Q mass spectrometer was equipped with an electrospray ion source. The mass spectrometer was operated in positive ion mode with a capillary voltage of 3.5 kV, an endplate offset of −500 V, nebulizer pressure of 1.8 bar, and N_2_ as te drying gas with a flow rate of 9.0 L/min at 200 °C. The mass spectra were recorded by means of a digitizer at a sampling rate of 2 GHz. The mass spectra were calibrated externally using the exact masses of clusters [(NaTFA)_n_ + TFA]^−^ from the solution of sodium trifluoroacetate (NaTFA). The spectra were evaluated with DataAnalysis 3.4 software from Bruker.

### 3.2. Chemical Synthesis

#### 3.2.1. 9-Decyl-3,6,12,15-Tetraoxa-9-Azaheptadecane-1,17-Diol (**6**)

1-Aminodecane **4** (1.6 mL, 8.0 mmol) was dissolved in dimethylformamide (25 mL), then powdered K_2_CO_3_ (4.0 g, 29 mmol, 3.6 equiv.), powdered KI (2.5 g, 15 mmol, 1.9 equiv.) and **5** (2.325 mL, 16 mmol, 2.0 equiv.) were added, and the reaction mixture was stirred overnight at 110 °C. The salts were filtered out from the cooled mixture, washed with dichloromethane and the solvent was evaporated. The residue was dissolved in dichloromethane (250 mL) and washed with aqueous 0.1 M NaOH (2 × 75 mL) and concentrated aqueous NaCl solution (75 mL). The organic phase was dried on Na_2_SO_4_ and filtered, and the solvent was evaporated in a vacuum. The product was purified by column chromatography (hexane/acetone 6:4 → 2:8 contained 0.2 *v*/*v*% triethylamine) to yield **6** (1.7 g, 50%) as a colorless syrup.

^1^H NMR (400 MHz, CDCl_3_) *δ* 3.75–3.69 (m, 4H, C*H*_2_), 3.68–3.54 (m, 16H, C*H*_2_), 3.29 (s, 2H, O*H*), 2.74 (t, 4H, J = 6.2 Hz, N-C*H*_2_), 2.55–2.47 (m, 2H, N-C*H*_2_), 1.44 (quin, *J* = 7.2, 6.6 Hz, 2H, C*H*_2_), 1.26 (s, 14H, C*H*_2_), 0.88 (t, 3H, *J* = 6.8 Hz, C*H*_3_). ^13^C NMR (100 MHz, CDCl_3_) *δ* 72.8 (2C, O-*C*H_2_), 70.5 (4C, O-*C*H_2_), 69.8, 61.8 (4C, O-*C*H_2_), 55.7, 54.0 (3C, N-*C*H_2_), 32.0, 29.8, 29.7, 29.4, 27,6, 26.9, 22.8 (8C, *C*H_2_), 14.2 (1C, *C*H_3_). MALDI-MS: *m/z* calculated for C_22_H_47_NO_6_ + Na^+^: 444.3296 [M + Na]^+^; found: 444.3291.

#### 3.2.2. N,N-Bis(2-(2-(2-Aminoethoxy)Ethoxy)Ethyl)Decan-1-Amine (**7**)

The solution of **6** (1.3 g, 3.08 mmol) in dry dichloromethane (5 mL) was cooled in an ice bath; then, *p*-toluenesulfonyl chloride (1.32 g, 6.9 mmol, 2.25 equiv.) and abs. pyridine (4.0 mL, dropwise) were added. The reaction mixture was stirred for two hours at 0–5 °C, then an additional 1.1 equiv. *p*-toluenesulfonyl chloride (629 mg, 3.3 mmol) was added. After one hour, dist. water (10 mL) was added to the reaction mixture, and it was stirred for 30 min. Then, the mixture was diluted with dichloromethane (200 mL) and washed with concentrated aqueous NaHCO_3_ solution (2 × 50 mL) and dist. water. (50 mL). The organic phase was dried on Na_2_SO_4_ and filtered, and the solvent was evaporated. The residue was purified by column chromatography (hexane/acetone 7:3 containing 1.0 *v*/*v*% triethylamine) to yield a yellowish syrup, which was used in the next step without characterization. The product of the previous reaction (1.49 g, 2.0 mmol) was dissolved in dimethylformamide (5 mL), and NaN_3_ (520 mg, 8.0 mmol) was added. The reaction mixture was stirred for 4 h at 90 °C, then the solvent was evaporated. The residue was dissolved in dichloromethane (200 mL) and washed with saturated aqueous NaHCO_3_ solution (2 × 50 mL), dist. water (50 mL) and concentrated aqueous NaCl solution (50 mL). The organic phase was dried on Na_2_SO_4_ and filtered, and the solvent was evaporated. The residue was purified by column chromatography (hexane/acetone 8:2 to 75:25 containing 1.0 *v*/*v*% triethylamine) to yield a yellowish syrup, which was used in the next step without characterization. This syrup (940 mg, 2.0 mmol) was dissolved in tetrahydrofurane (10 mL), then triphenylphosphine (2.62 g, 10 mmol, 5 equiv.) was added, and the mixture was stirred at 50 °C. After gassing ceased, it was stirred for another two hours, dist. water (2 mL) was added, and the reaction mixture was stirred overnight at room temperature. Then, the solvent was evaporated, and the product was purified by flash column chromatography (dichloromethane/methanol 7:3 then dichloromethane/methanol/concentrated aqueous ammonia solution 7:3:0.5) to yield **7** (750 mg, 58% for three steps) as a colorless syrup.

^1^H NMR (360 MHz, CDCl_3_) *δ* 3.61 (s, 8H, O-C*H*_2_), 3.59–3.47 (m, 8H, O-C*H*_2_), 2.87 (t, 4H, *J* = 5.2 Hz, N-C*H*_2_), 2.72, (t, 4H, *J* = 6.4 Hz, N-C*H*_2_), 2.55–2.44 (m, 2H, N-C*H*_2_), 1.51 (s, 4H, N*H*_2_), 1.43 (quin, 2H, *J* = 7.3, 6.6, 6.6 Hz, C*H*_2_), 1.26 (s, 14H, C*H*_2_), 0.88 (t, 3H, *J* = 6.6 Hz, C*H*_3_). ^13^C NMR (90 MHz, CDCl_3_) *δ* 73.5, 70.4, 70.4, 69.9 (8C, O-*C*H_2_), 55.6 (1C, N-*C*H_2_), 54.1 (2C, N-*C*H_2_), 41.8 (2C, *C*H_2_-NH_2_), 31.9, 29.7, 29.6, 29.4, 27.5, 27.1, 22.7 (8C, *C*H_2_), 14.2, (1C, *C*H_3_). MALDI-MS: *m/z* calculated for C_22_H_49_N_3_O_4_ + Na^+^: 442.3615 [M + Na]+; found: 442.3613.

#### 3.2.3. N,N-Bis(2-(2-(2-Isothiocyanatoethoxy)Ethoxy)Ethyl)Decan-1-Amine (**8**)

Compound **7** (700 mg, 1.67 mmol) was dissolved in abs. ethanol (2 mL) in a round bottom flask equipped with a calcium chloride drying tube. CS_2_ (2.0 mL, 16.7 mmol, 10 equiv.) and Et_3_N (466 μL, 3.34 mmol, 2 equiv.) were added, and the mixture was stirred for 30 min. After cooling it to 0 °C, di-*tert*-butyl dicarbonate (726 mg, 3.34 mmol, 2 equiv.) dissolved in abs. ethanol (1 mL) and 4-dimethylaminopyridine (20 mg) dissolved in abs. ethanol (0.5 mL) were added. The reaction mixture was stirred for 1 h at room temperature, then the solvent was evaporated, and the product was purified by flash column chromatography (dichloromethane/acetone 7:3 containing 0.1 *v*/*v*% Et_3_N) to yield **8** (640 mg, 76%) as a yellow syrup.

^1^H NMR (360 MHz, CDCl_3_) *δ* 3.76–3.60 (m, 16H, OC*H*_2_), 3.56 (t, 4H, *J* = 6.3 Hz), 2.73 (t, 4H, *J* = 6.3 Hz, NC*H*_2_), 2.56–2.44 (m, 2H, NC*H*_2_), 1.43 (quin, 2H, *J* = 6.7 Hz, C*H*_2_), 1.23 (s, 14H, C*H*_2_), 0.88 (t, 3H, *J* = 6.8 Hz, C*H*_3_). ^13^C NMR (90 MHz, CDCl_3_) *δ* 70.9, 70.6, 70.0, 69.4, (8C, O*C*H_2_), 55.7 (1C, N*C*H_2_), 54.1 (2C, N*C*H_2_), 45.3 (2C, *C*H_2_-NCS), 32.0, 29.7, 29.4, 27.5, 27,2, 22.7 (8C, *C*H_2_), 14.2 (1C, *C*H_3_). MALDI-MS: *m/z* calculated for C_24_H_45_N_3_O_4_S_2_ + H^+^: 504.2924 [M + H]+; found: 504.2937.

#### 3.2.4. Compound **12**

To the solution of **2** (120 mg, 0.077 mmol) in dimethylformamide (1.5 mL), Et_3_N (21.5 μL, 0.154 mmol, 2 equiv.) and **8** (193 mg, 0.38 mmol, 5 equiv.) were added, and the reaction mixture was stirred for 3 h; then, the product was precipitated with diethyl ether and filtered and washed with diethyl ether. After drying, it was dissolved in dimethylformamide (2 mL), then Et_3_N (11.2 μL, 0.08 mmol) and **2** (80 mg, 0.051 mmol) were added and the reaction mixture was stirred for 20 h. The product was precipitated with diethyl ether, filtered and washed with diethyl ether, and then purified by column chromatography (acetonitrile/H_2_O 85:15 → 80:20 → 75:25 → 70:30) to yield **12** (88 mg, 32% for two steps) as a white powder. NMR data of **12** are given in Table 2.

HPLC-MS: t_R_ = 16.76 min. ESI-MS: *m/z* calculated for C_168_H_181_Cl_4_N_19_O_60_S_2_ + 3H^+^: 1211.671 [M + 3H]^3+^; found: 1211.668.

#### 3.2.5. Compound **13**

Compound **9** (126 mg, 0.075 mmol) was dissolved in dimethylformamide (1.5 mL), then Et_3_N (12.5 μL, 0.09 mmol, 1.2 equiv.) and **8** (227 mg, 0.45 mmol, 6 equiv.) were added, and the mixture was stirred for 3 h. The intermediate product (**11**) was precipitated with diethyl ether, filtered, washed with ether and dried in a vacuum. Then it was dissolved in dimethylformamide (2 mL), then Et_3_N (12.5 μL, 0.09 mmol, 1.2 equiv.) and **9** (113 mg, 67.5 μmol, 0.9 equiv.) were added. After stirring for 20 h, the product was purified by column chromatography (acetonitrile/H_2_O 8:2 → 7:3 containing 0.1 *v*/*v*% AcOH →7:3 → 6:4 → 1:1 containing 1.0 *v*/*v*% AcOH), then by column chromatography using Sephadex LH-20 as a stationary phase (acetonitrile/H_2_O 1:1) to yield **13** (74 mg, 26% for two steps) as a white powder. NMR data of **13** are given in Table 3.

ESI-MS: *m/z* calculated for C_182_H_213_Cl_4_N_23_O_58_S_2_ + 3H^+^: 1286.429 [M + 3H]^3+^; found: 1286.414.

#### 3.2.6. Compound **14**

To the solution of **2** (360 mg, 0.23 mmol) in dimethylformamide (3 mL), Et_3_N (64 μL, 0.46 mmol, 2 equiv.) and di-tert-butyl dicarbonate (55 mg, 0.253 mmol, 1.1 equiv.) in dimethylformamide (1 mL) were added. After two hours stirring, the intermediate product was precipitated with diethyl ether, filtered and purified by column chromatography (acetonitrile/H_2_O 90:10 → 85:15 containing 0.2 *v*/*v*% AcOH). The intermediate product (285 mg (0.17 mmol) was dissolved in a mixture of DMF (1.5 mL) and DMSO (1.5 mL) and ethylenediamine (23 μL, 0.34 mmol, 2 equiv.) was added. After 5 h of stirring, ethyl acetate (40 mL) and diethyl ether (20 mL) were added, and the precipitate was filtered and washed with diethyl ether. The product was purified by column chromatography (acetonitrile/H_2_O = 90:10 → 85:15 containing 0.2 *v*/*v*% AcOH → 80:20 → 75:25 → 70:30 containing 0.3% *v*/*v*% AcOH) to yield **14** (130 mg, 35% for two steps).

MALDI-MS: *m/z* calculated for C_79_H_82_Cl_2_N_10_O_29_ + Na^+^: 1727.452 [M + Na]^+^; found: 1727.565.

#### 3.2.7. Compound **16**

Compound **14** (65 mg, 38 μmol) was dissolved in dimethylformamide (0.8 mL), then Et_3_N (10.6 μL, 76 μmol, 2 equiv.) and **8** (55 μL, 0.11 mmol, 3 equiv.) were added. After 3 h of stirring, the intermediate product was precipitated with diethyl ether, filtered, washed with diethyl ether and dried. The intermediate product (**15**) (62 mg, ~25 μmol, ~90% purity) was dissolved in dimethylformamide (1 mL), then Et_3_N (7 μL, 50 μmol, 2 equiv.) and **2** (39 mg, 25 μmol, 1 equiv.) were added and the reaction mixture was stirred for 20 h. Diethyl ether was added, and the precipitate was filtered and washed with diethyl ether. The precipitate was purified by column chromatography (acetonitrile/H_2_O 9:1 → 85:15) and then by column chromatography using Sephadex LH-20 as a stationary phase (H_2_O/acetonitrile 70:30 → 65:35 → 60:40 → 58:42). The intermediate product (46 mg) was dissolved in trifluoroacetic acid (1 mL), and the mixture was stirred for 1 h. The solvent was evaporated, and the product was purified by column chromatography (acetonitrile/H_2_O 80:20 →75:25 containing 0.2 *v*/*v*% AcOH) to yield **16** (31 mg, 22% for three steps) as a white powder.

HPLC-MS: t_R_ = 9.89 min. ESI-MS: *m/z* calculated for C_170_H_187_Cl_4_N_21_O_59_S_2_ + 3H^+^: 1225.691 [M + 3H]^3+^; found: 1225.693.

#### 3.2.8. Compound **17**

Compound **14** (65 mg, 38 μmol) was dissolved in dimethylformamide (0.8 mL), then Et_3_N (10.6 μL, 76 μmol, 2 equiv.) and **8** (55 μL, 0.11 mmol, 3 equiv.) were added. After 3 h of stirring, the intermediate product was precipitated with diethyl ether, filtered, washed with diethyl ether and dried. The intermediate product (**15**) (65 mg, ~26 μmol, ~90% purity) was dissolved in dimethylformamide (1 mL), then Et_3_N (7 μL, 50 μmol, 2 equiv.) and **9** (44 mg, 26 μmol, 1 equiv.) were added and the reaction mixture was stirred for 20 h. Diethyl ether was added, the precipitate was filtered and washed with diethyl ether, then purified by column chromatography (acetonitrile/H_2_O 85:15 → 80:20 containing 0.2 *v*/*v*% AcOH). The intermediate product (45 mg) was dissolved in trifluoroacetic acid (1 mL), and the mixture was stirred for 1 h. The solvent was evaporated, and the product was purified by column chromatography (acetonitrile/H_2_O 8:2 →7:3 → 6:4 containing 0.5 *v*/*v*% AcOH) to yield **17** (21 mg, 15% for three steps) as a white powder.

HPLC-MS: t_R_ = 7.76 min. ESI-MS: *m/z* calculated for C_177_H_203_Cl_4_N_23_O_58_S_2_ + 3H^+^: 1263.070 [M + 3H]^3+^; found: 1263.062.

#### 3.2.9. Compound **20**

Compound **18** (50 mg, 0.14 mmol) was dissolved in abs. dimethylformamide (2 mL) and *N*-hydroxysuccinimide (NHS) (0.154 mmol, 18 mg, 1.1 equiv) and 1-ethyl-3-(3′-dimethylaminopropyl)carbodiimide·HCl (EDC·HCl) (0.154 mmol, 29.5 mg, 1.1 equiv) were added and stirred for 6 h. Teicoplanin pseudoaglycone **3** (0.071 mmol, 100 mg) was dissolved in 1 mL of dimethylformamide and the solution was added to the previously prepared mixture containing the active ester of **19**. The reaction mixture was stirred overnight, then the solvent was evaporated, and the residue was purified by flash column chromatography (acetonitrile/H_2_O 10:0 → 95:1 → 9:1). The product was dissolved in trifluoroacetic acid (1 mL), and the mixture was stirred for 1 h. The trifluoroacetic acid was evaporated, then 2 mL of toluene was added and evaporated from the product to yield **20** (95 mg, 87%) as a white powder.

ESI-MS: *m/z* calculated for C_72_H_63_Cl_2_N_11_O_24_^2−^: 767.670 [M-2H]^2−^; found: 767.657.

## 4. Conclusions

In this work, we have reported on the first synthesis of covalent and complex dimers of teicoplanin derivatives. We have found that either covalent or complex dimer formation of teicoplanin pseudoaglycone derivatives proved to be ineffective for improving the antibacterial activity of the monomeric parent compounds. This phenomenon is different from the dimerization effect of vancomycin derivatives, showing the different mechanism of the antibacterial action of the two subgroups of the glycopeptide antibiotic family.

Importantly, although several results can be obtained on the effect of dimerization of vancomycin [9,19,20,21,22,23,24,25,26,27,28,29], eremomycin [11], ristocetin [14] and dalbavancin [12] on antibacterial activity, our study presented here provided the first experimental data on the relationship between the dimerization and antibacterial effects of teicoplanin. Moreover, the new methodologies used for dimer formation, i.e., a linker chain with lipophilic substituent and cobalt complex formation, can be applied to other glycopeptide antibiotic derivatives.

## Figures and Tables

**Table 1:** Antibacterial effect.

Bacterial Strains.	Teico (1)	2	3	Co (acac)	12	13	16	17	20	21
	MIC ^[g]^ (μg/mL)									
*Bacillus subtilis* ATCC ^[a]^ 6633	0.5	2	2	256	64	4	4	8	2	4
*Staphylococcus aureus* MSSA ^[b]^ ATCC 29213	0.5	4	2	256	64	8	16	64	4	4
*Staphylococcus aureus* MRSA ^[c]^ ATCC 33591	0.5	2	1	256	256	4	8	16	4	4
*Staphylococcus epidermidis* ATCC 35984 biofilm	2	2	2	256	16	4	16	8	4	4
*Staphylococcus epidermidis* mecA ^[d]^	16	4	1	256	64	4	64	16	2	2
*Enterococcus faecalis* ATCC 29212	2	8	4	256	4	8	16	16	4	8
*Enterococcus faecalis* 15376 VanA ^[e]^	256	4	256	256	16	4	8	8	4	8
*Enterococcus faecalis* ATCC 51299 VanB ^[f]^	4	8	2	256	32	8	32	16	4	4

^[a]^ American Type Culture Collection. ^[b]^ Methicillin-sensitive *Staphylococcus aureus*. ^[c]^ Methicillin-resistant *Staphylococcus aureus*. ^[d]^ mecA gene expression in *Staphylococcus*. ^[e]^ vanA gene positive. ^[f]^ vanB gene positive. ^[g]^ Minimum inhibitory concentration.

**Table 2:** NMR data of **12**.

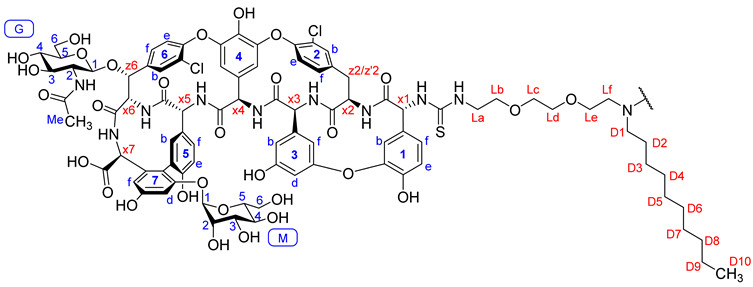					
**^13^C Shift (ppm)**	**^1^H Shift (ppm)**	**Annotation**	**^13^C Shift (ppm)**	**^1^H Shift (ppm)**	**Annotation**
96.3	5.26	M1	75.1	5.33	z6
98.3	4.35	G1	60.6	4.14	x6
104.4	5.08	4f	43.5	3.58	La
107.7	5.62	4b	56.0	3.35	G2
100.7	6.71	7d	60.4	3.50	M6
104.4	6.34	3f	60.8	3.68	G6
105.7	6.50	3d	60.8	3.47	G6
107.6	6.49	7f	53.2	2.59	Lf
110.7	6.34	3b	54.2	2.43	D1
115.7	6.72	5e	69.4	3.49	Lb-d
116.2	6.68	1b	68.8	3.43	Le
117.7	6.94	1e	69.6	3.26	M3
123.1	7.26	6e	70.4	3.23	M2
123.2	7.05	1f	70.3	3.08	G4
125.6	6.73	5f	72.7	3.44	G3
124.5	7.26	2e	73.4	3.49	M5
128.0	7.25	6f	76.6	3.05	G5
129.8	7.60	2f	65.7	3.48	M4
128.4	7.81	6b	36.3	3.29	z2/z’2
130.7	7.22	2b	36.4	2.81	z2/z’2
135.1	7.02	5b	22.9	1.88	G-Me
54.4	5.65	x4	21.8	1.24	D9
60.0	6.02	x1	26.5	1.34	D2
57.8	5.25	x3	26.4	1.21	D3
54.1	4.85	x2	28.6	1.22	D4–7
53.3	4.40	x5	31.0	1.23	D8
56.6	4.47	x7	13.6	0.85	D10

**Table 3:** NMR data of **13**.

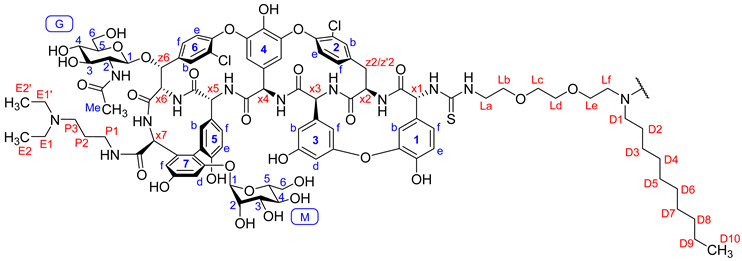					
**^13^C Shift (ppm)**	**^1^H Shift (ppm)**	**Annotation**	**^13^C Shift (ppm)**	**^1^H Shift (ppm)**	**Annotation**
60.1	6.01	x1	23.0	1.91	G-Me
54.1	4.86	x2	26.2	1.64	P2
57.9	5.27	x3	49.8	2.46	P3
54.3	5.69	x4	37.2	3.19	P1
53.4	4.40	x5	54.4	2.43	D1
56.7	4.47	x7	26.5	1.34	D2
60.8	4.16	x6	26.4	1.22	D3
74.9	5.42	z6	28.7	1.25	D4–7
61.2	3.73	G6	31.0	1.23	D8
61.2	3.42	G6	21.7	1.26	D9
96.4	5.25	M1	45.9	2.51	E1/E1′
98.3	4.34	G1	135.2	7.12	5b
104.3	5.11	4f	125.6	6.74	5f
107.4	5.62	4b	100.6	6.72	7d
55.9	3.29	G2	107.6	6.41	7f
76.5	3.05	G5	110.4	6.34	3b
60.5	3.52	M6	104.1	6.33	3f
70.4	3.19	M2	105.5	6.50	3d
70.6	2.99	G4	116.0	6.73	5e
69.7	3.22	M3	123.3	7.02	1f
72.5	3.49	G3	116.3	6.67	1b
73.5	3.48	M5	118.0	6.92	1e
65.8	3.50	M4	123.0	7.29	6e
69.3	3.51	Lb-d	128.5	7.82	6b
68.9	3.43	Le	129.8	7.61	2f
43.5	3.59	La	130.8	7.23	2b
53.2	2.59	Lf	124.5	7.28	2e
11.1	0.98	E2/E2′	127.8	7.26	6f
13.8	0.85	D10			

## Data Availability

The data presented in this study are available in article and supplementary material.

## Supplementary Materials

The following are available online at https://www.mdpi.com/article/10.3390/ph15010077/s1, NMR spectra of **6**, **7**, and **8**, MS spectra of compounds **6**, **7**, **13**, **14** and **20**, HSQC spectra of **12** and **13**, and HPLC-MS chromatograms of compounds **12**, **16** and **17**.
