# Supplementary material for: The First Dimeric Derivatives of the Glycopeptide Antibiotic Teicoplanin
Source: Pharmaceuticals (Basel). 2022 Jan 8;15(1):77. doi: 10.3390/ph15010077 (PMC8779952; doi:10.3390/ph15010077)

SUPPLEMENTARY INFORMATION

The first dimeric derivatives of the glycopeptide antibiotic teicoplanin

Ilona Bereczki, Zsolt Szűcs, Gyula Batta, Tamás Milán Nagy, Eszter Ostorházi,
Katalin E. Kövér, Anikó Borbás and Pál Herczegh

Table of content

NMR and MS spectra of compound **6** 3

NMR and MS spectra of compound **7** 4

NMR spectra of compound **8** 6

HSQC spectra (400 MHz) and HPLC-MS of compound **12** 7

HSQC (400 MHz) and MS spectra of compound **13** 9

MS spectrum of compound **14** 11

HPLC-MS of compound **16** 12

HPLC-MS of compound **17** 13

MS spectrum of compound **20** 14

NMR and MS spectra of compound **6**

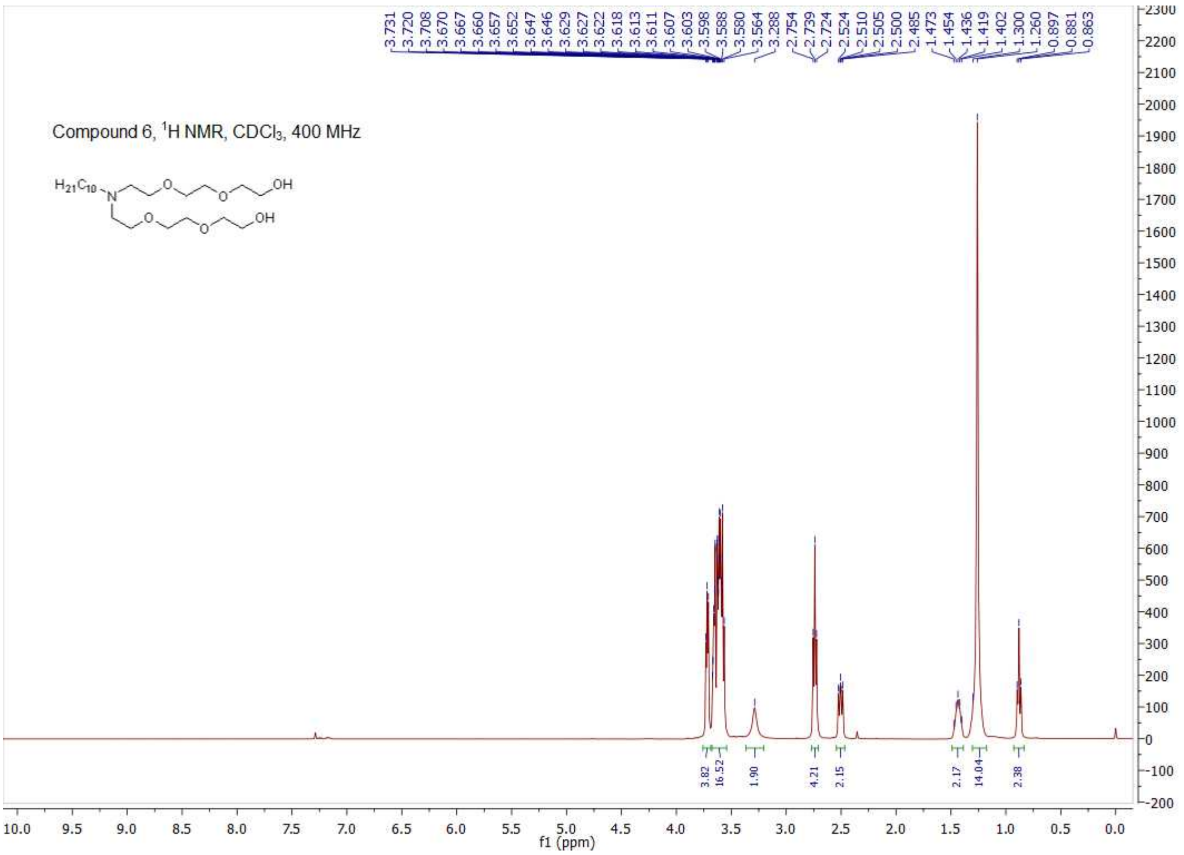

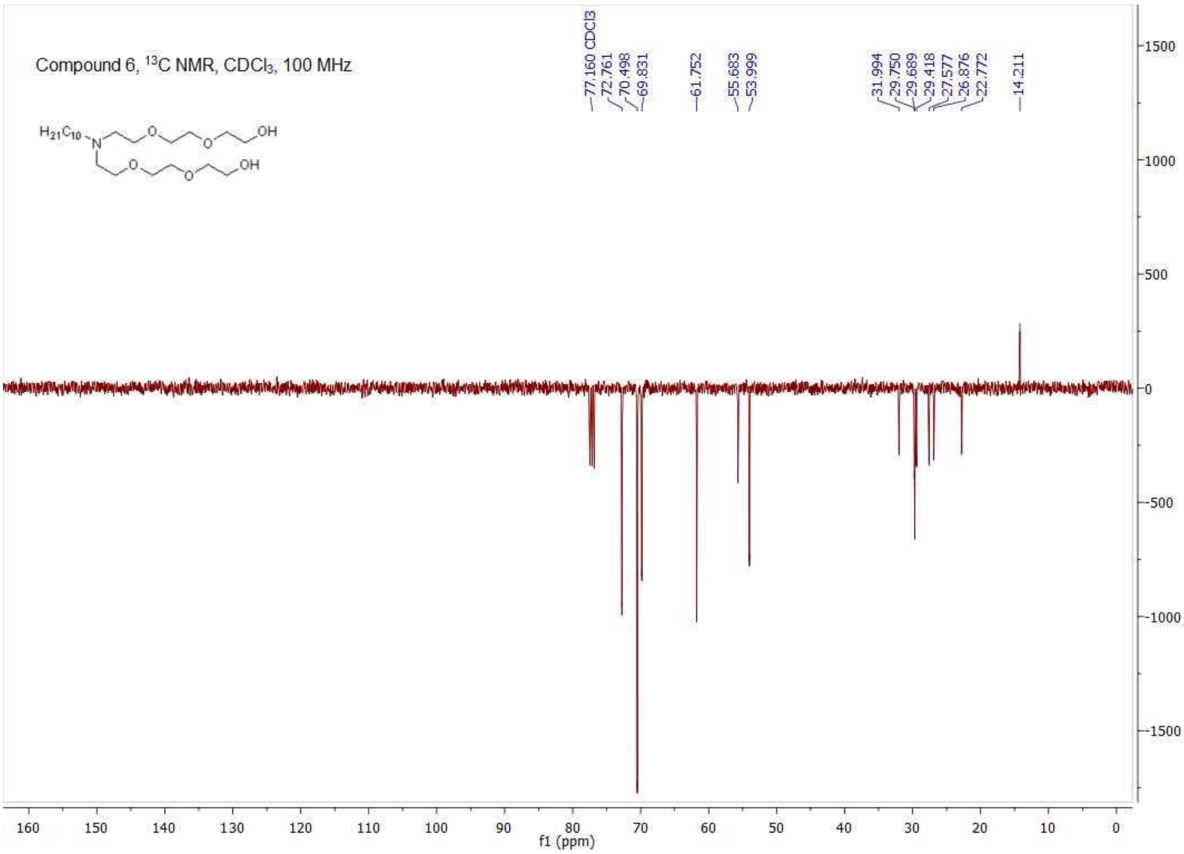

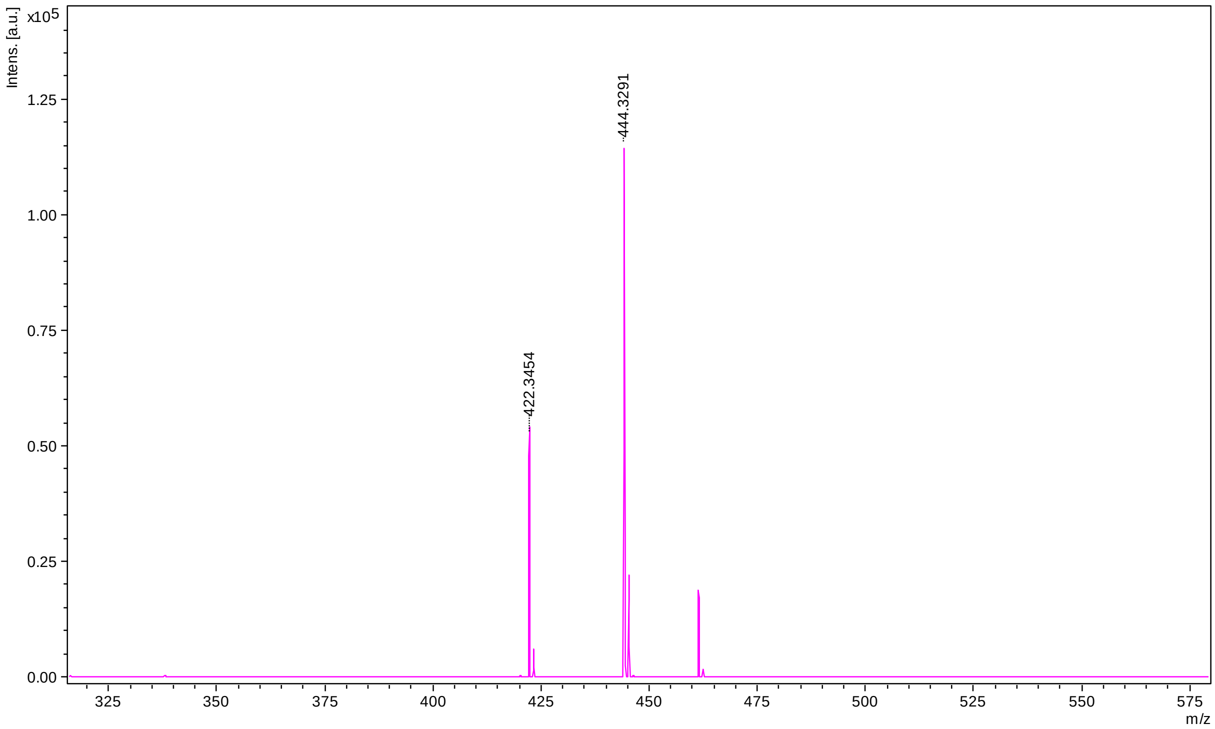

NMR and MS spectra of compound **7**

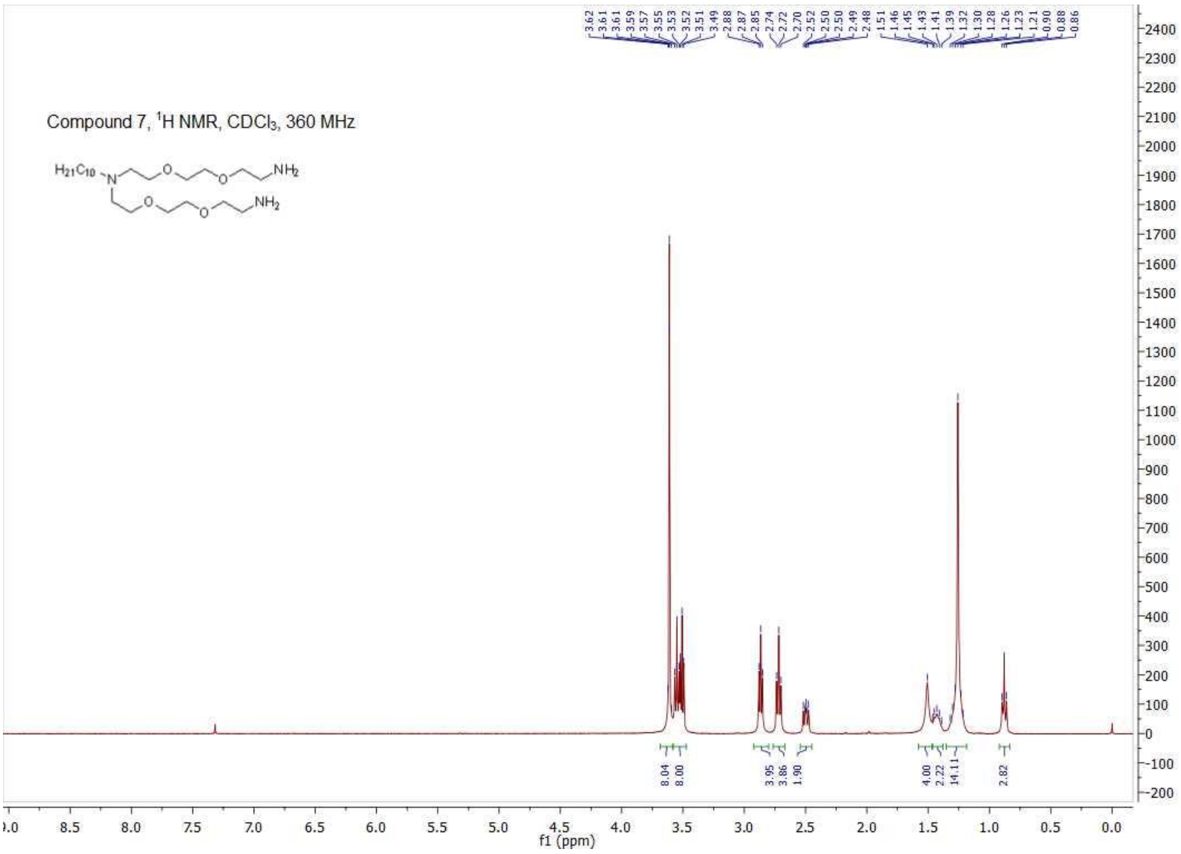

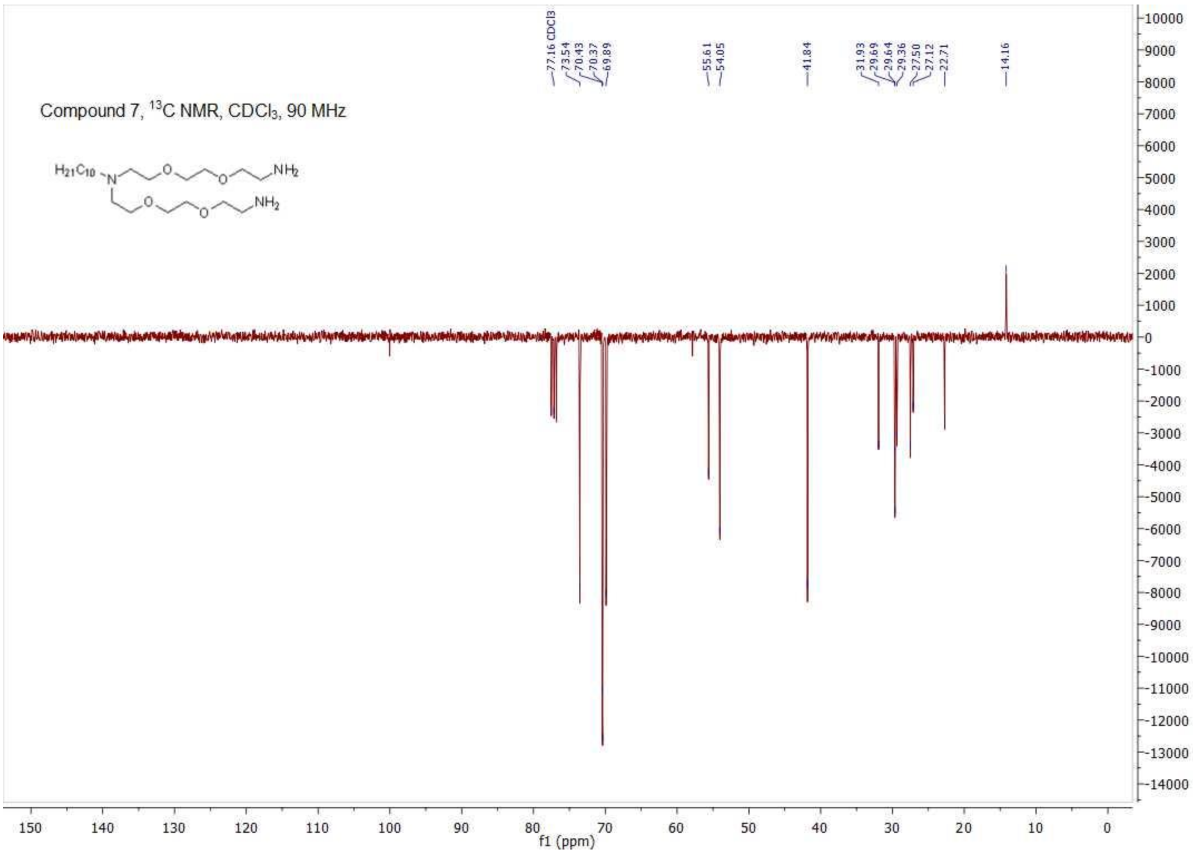

Compound 7, ^{13}C NMR, CDCl$_3$, 90 MHz
77.16 CDCl3
73.54
70.43
70.37
69.89
55.61
54.05
41.84
31.93
29.69
29.64
29.36
27.50
27.12
22.71
14.16
f1 (ppm)

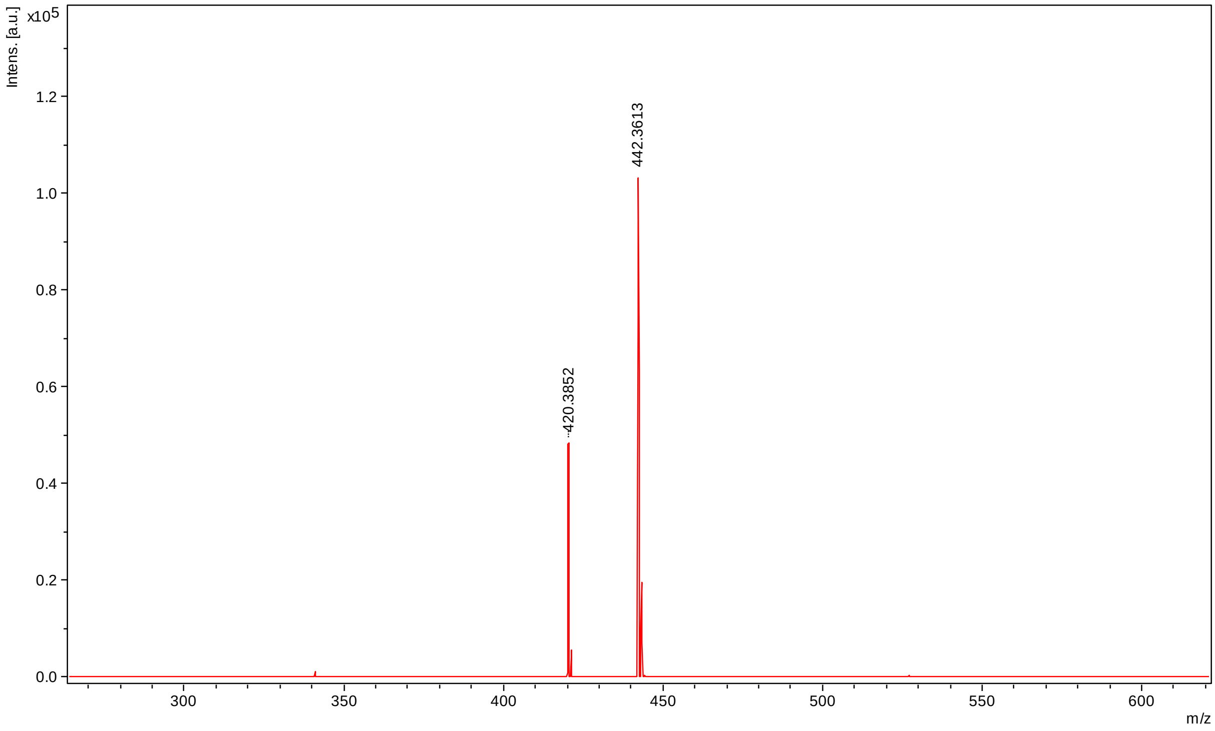

Intens. [a.u.]
x10^5
420.3852
442.3613
m/z

NMR spectra of compound **8**

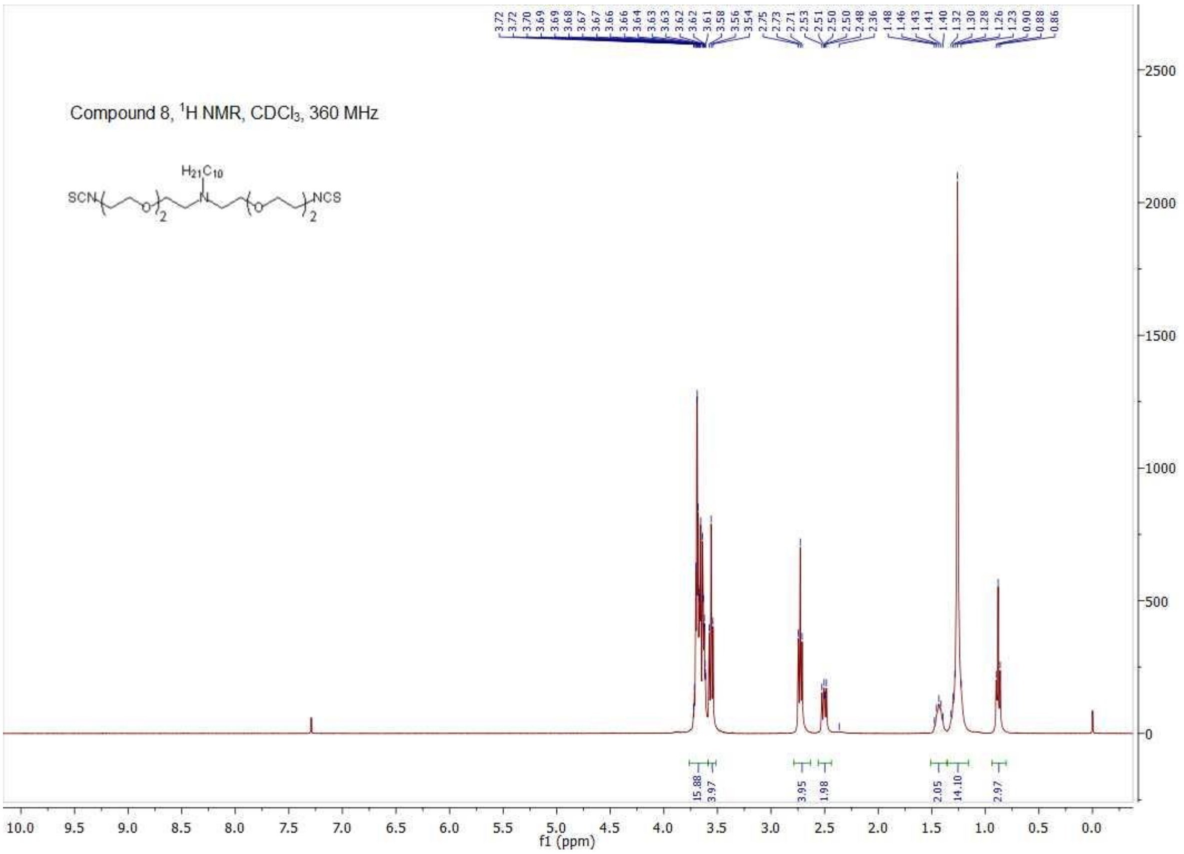

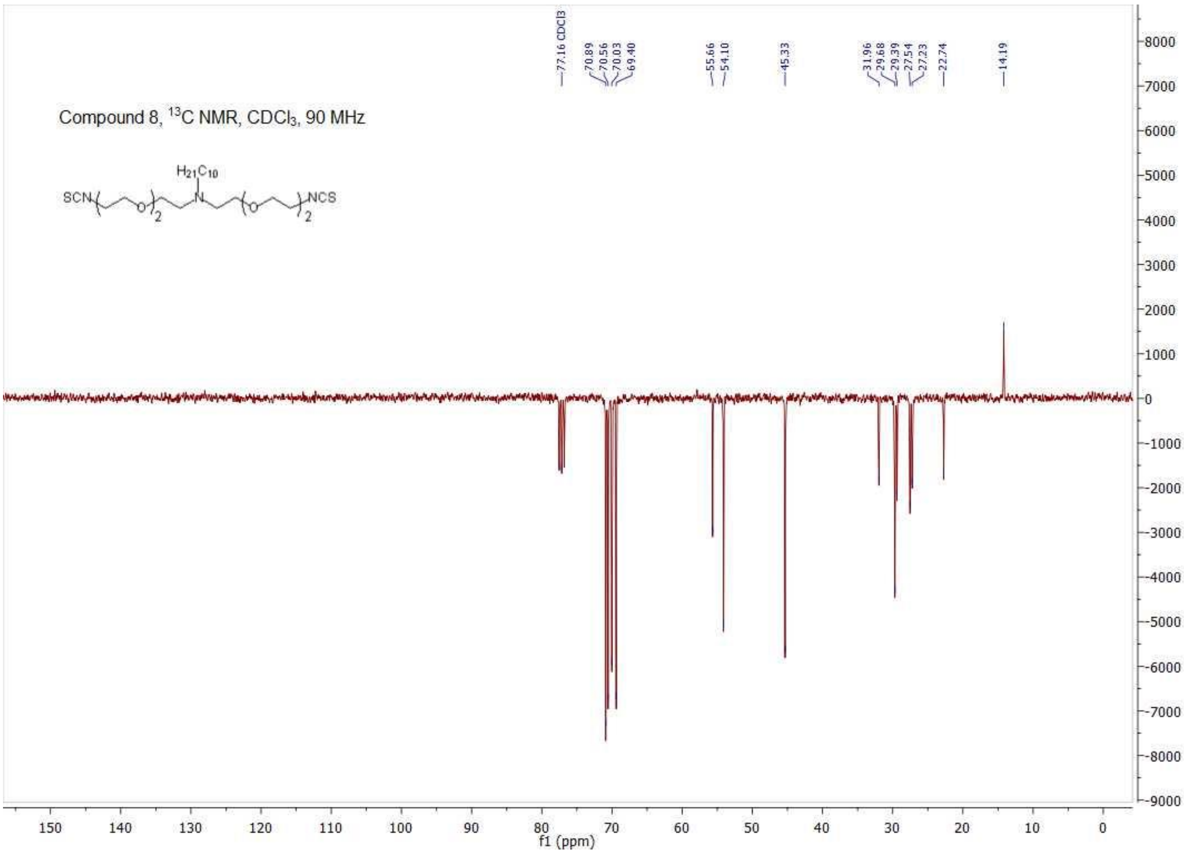

HSQC spectra (400 MHz) and HPLC-MS of compound **12**

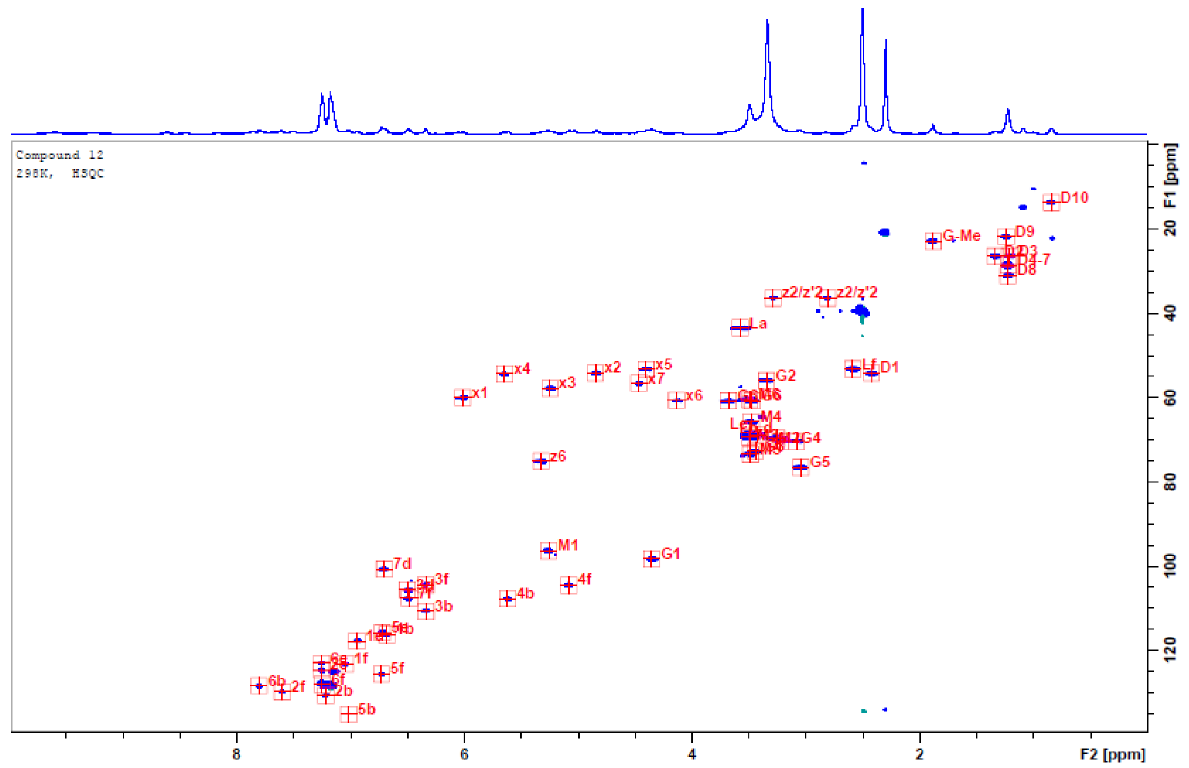

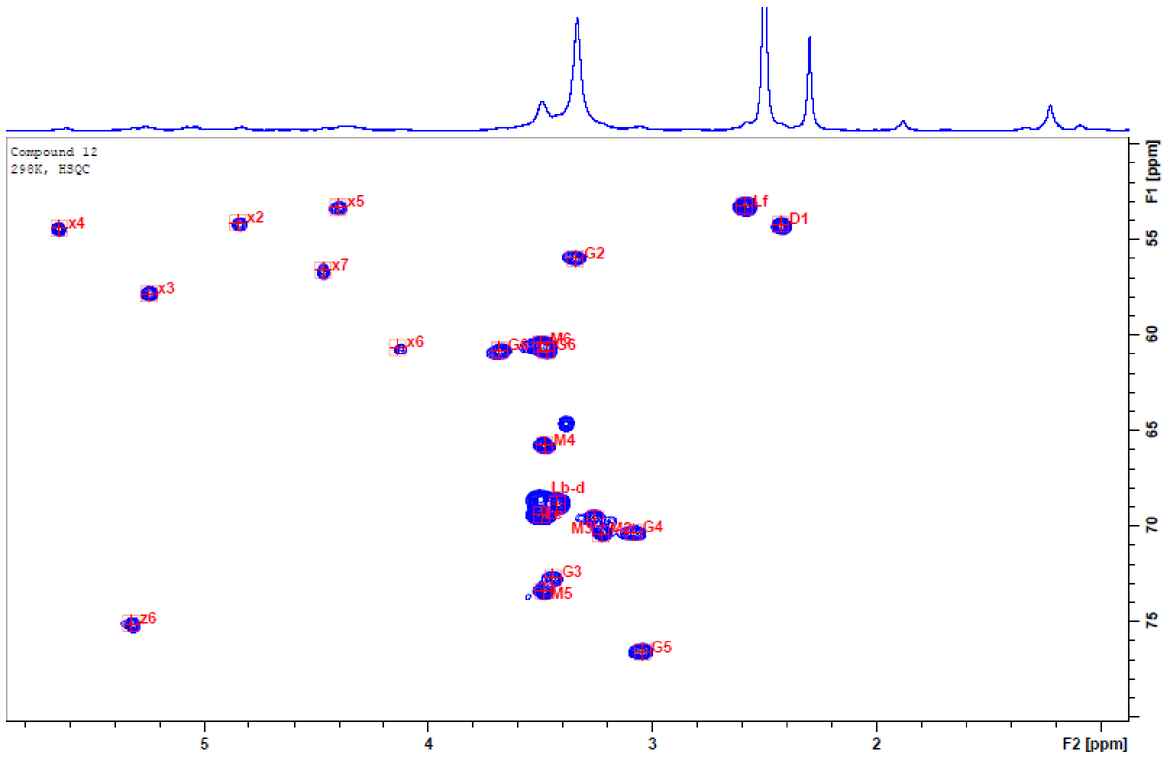

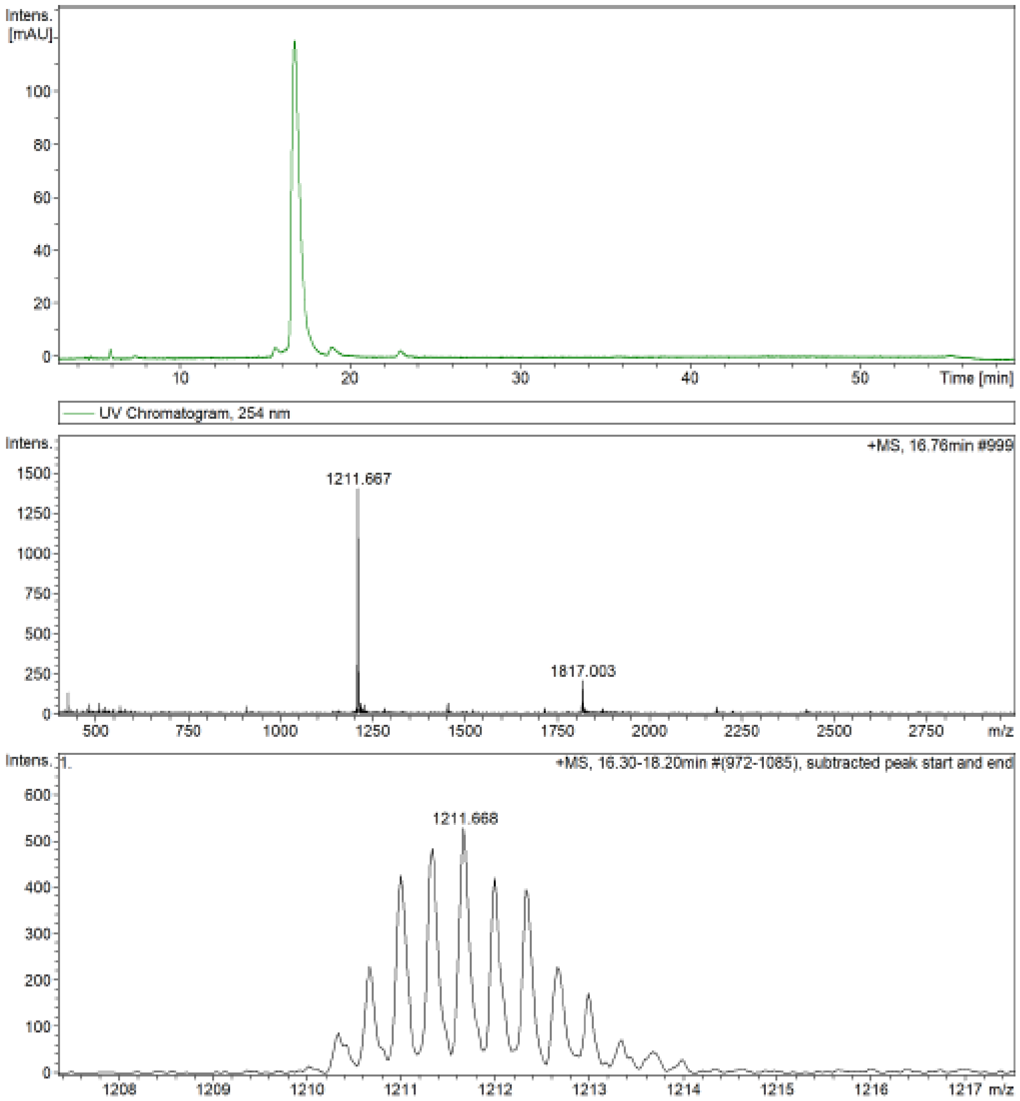

Intens.
[mAU]
100
80
60
40
20
0
10
20
30
40
50
Time [min]
UV Chromatogram, 254 nm
Intens.
+MS, 16.76min #999
1211.667
1817.003
1500
1250
1000
750
500
250
0
500
750
1000
1250
1500
1750
2000
2250
2500
2750
m/z
Intens.
+MS, 16.30-18.20min #(972-1085), subtracted peak start and end
1211.668
600
500
400
300
200
100
0
1208
1209
1210
1211
1212
1213
1214
1215
1216
1217
m/z

HSQC (400 MHz) and MS spectra of compound **13**

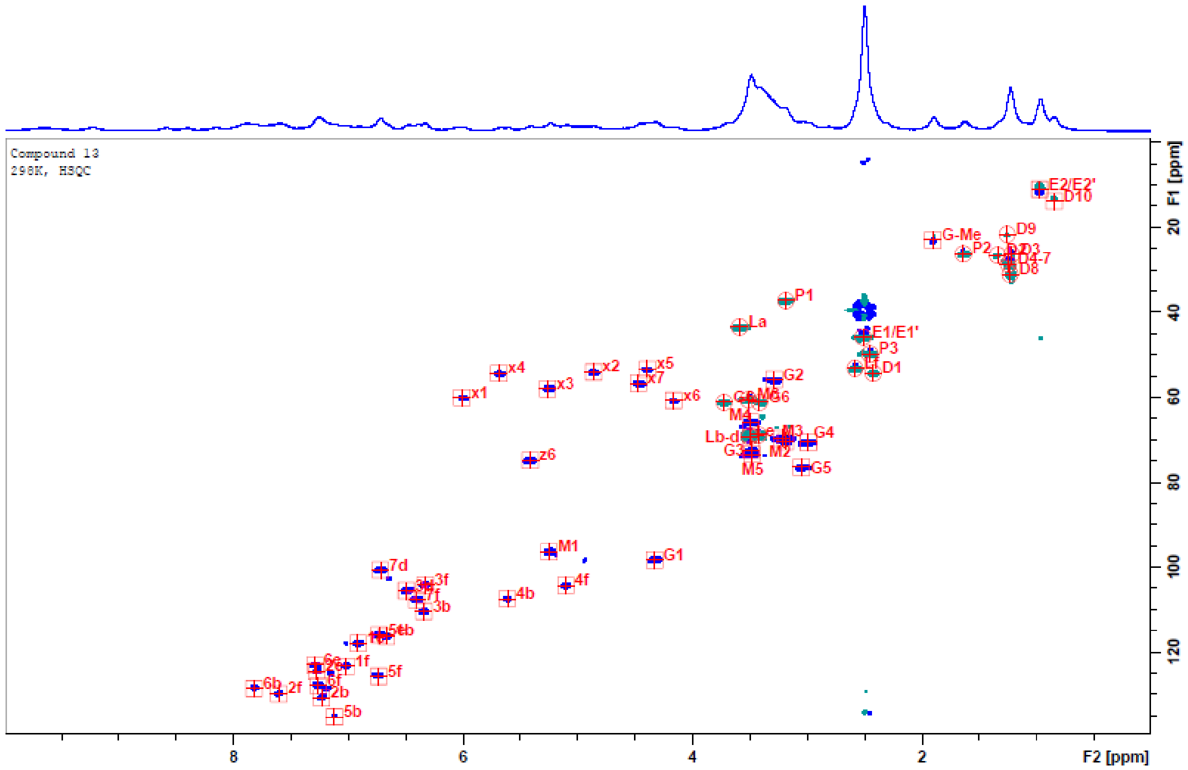

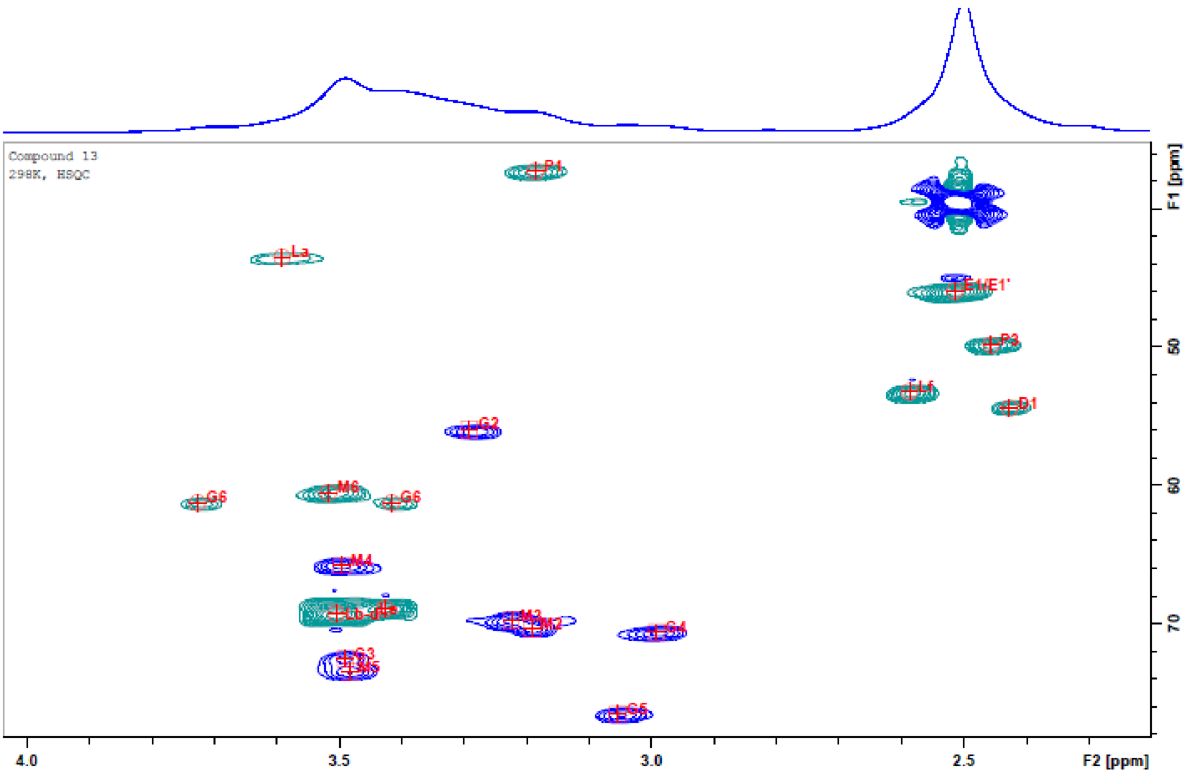

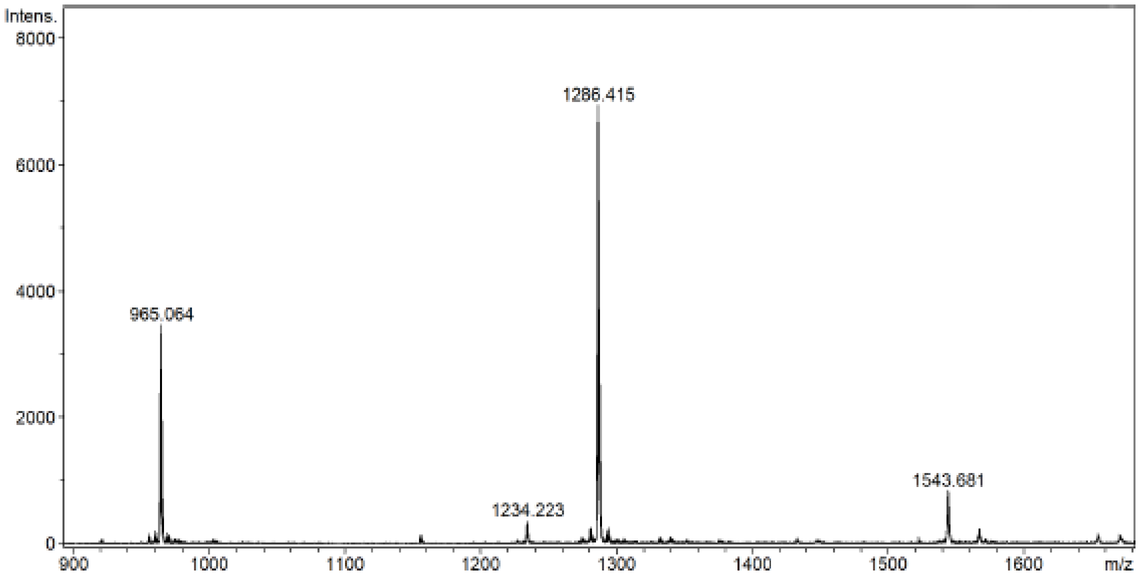
Intens.
8000
6000
4000
2000
0
1286.415
965.064
1234.223
1543.681
900
1000
1100
1200
1300
1400
1500
1600
m/z

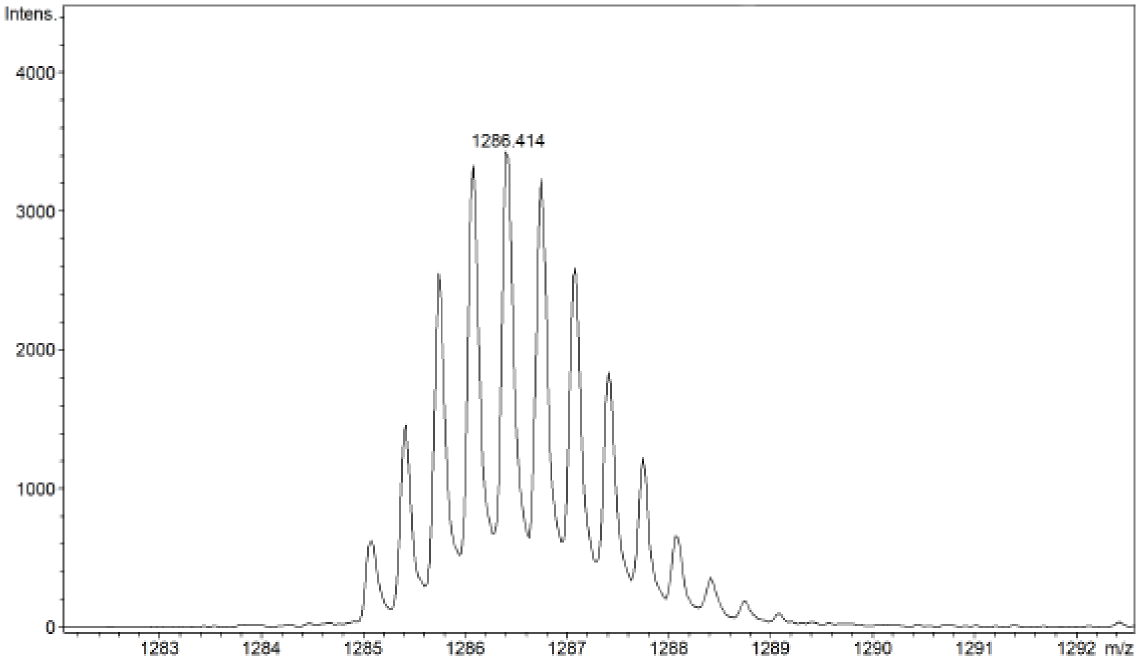
Intens.
4000
3000
2000
1000
0
1286.414
1283
1284
1285
1286
1287
1288
1289
1290
1291
1292
m/z

MS spectrum of compound **14**

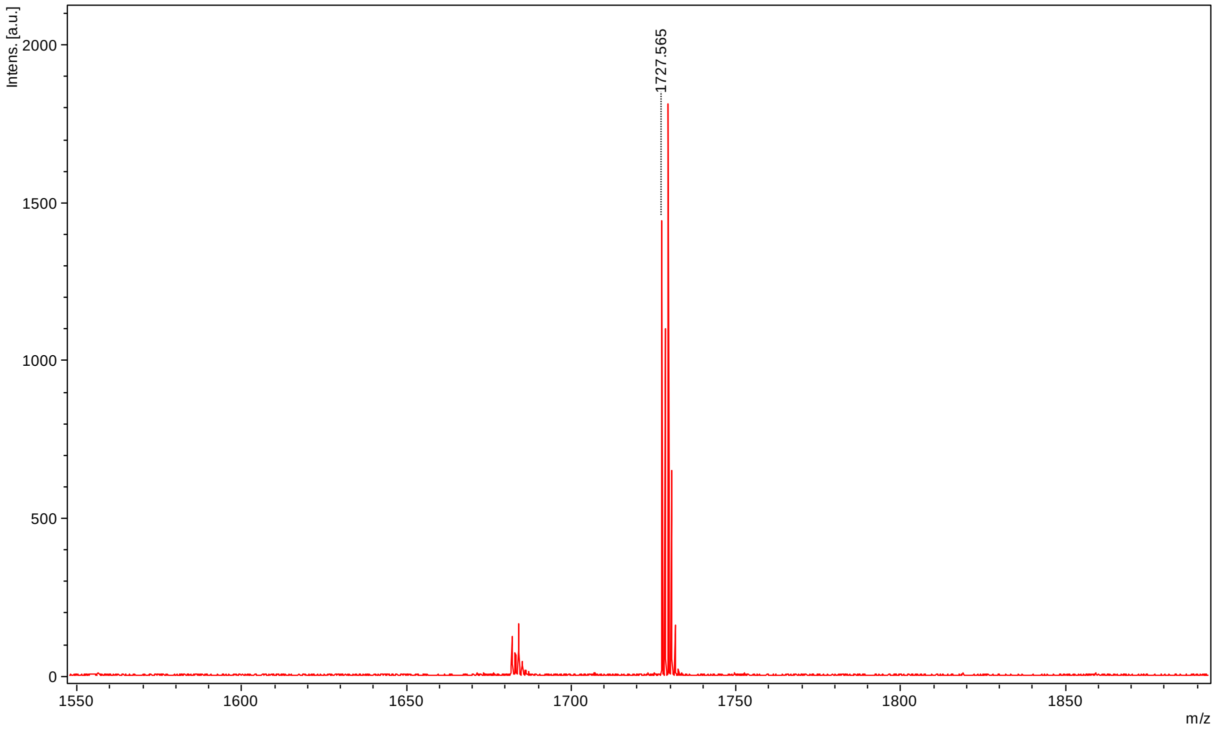

HPLC-MS of compound **16**

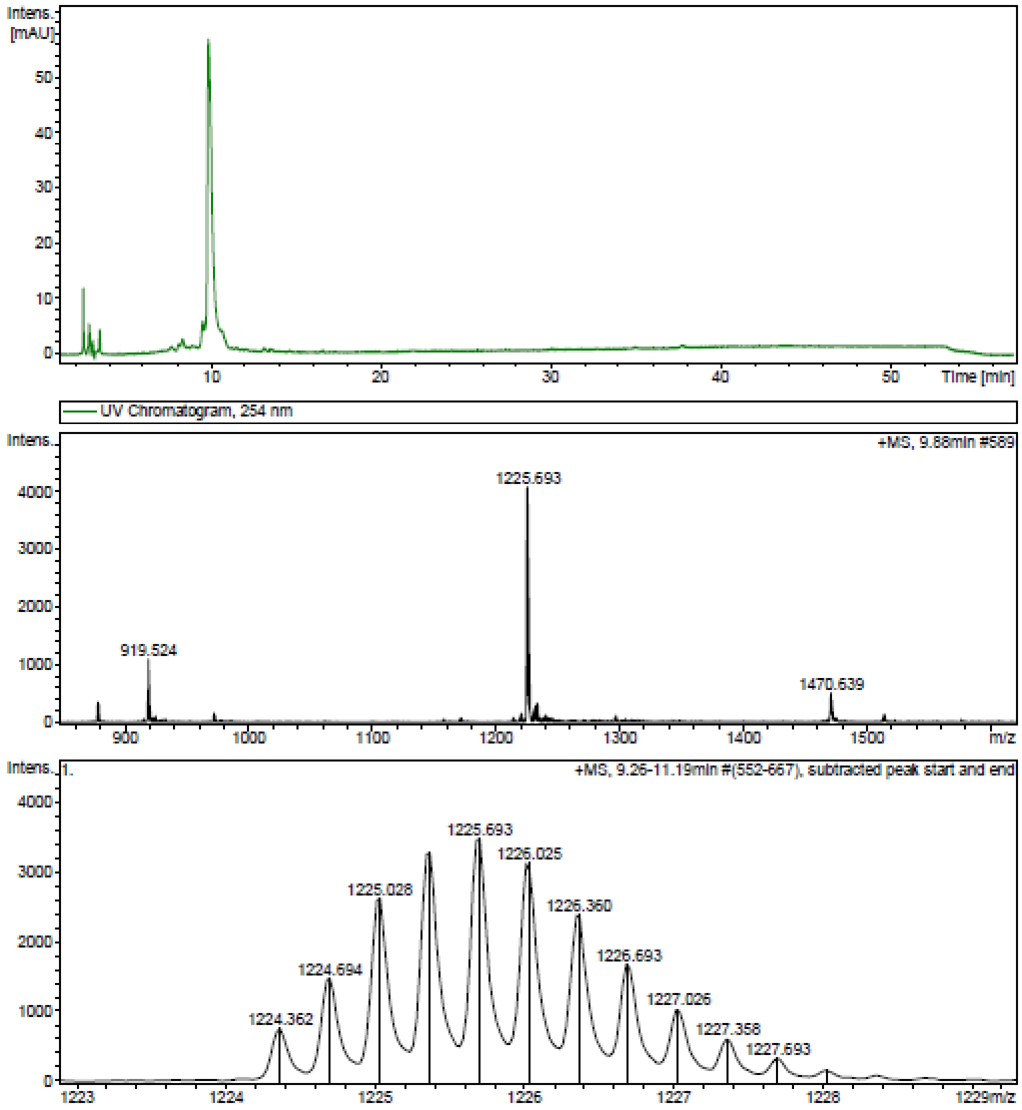

HPLC-MS of compound **17**

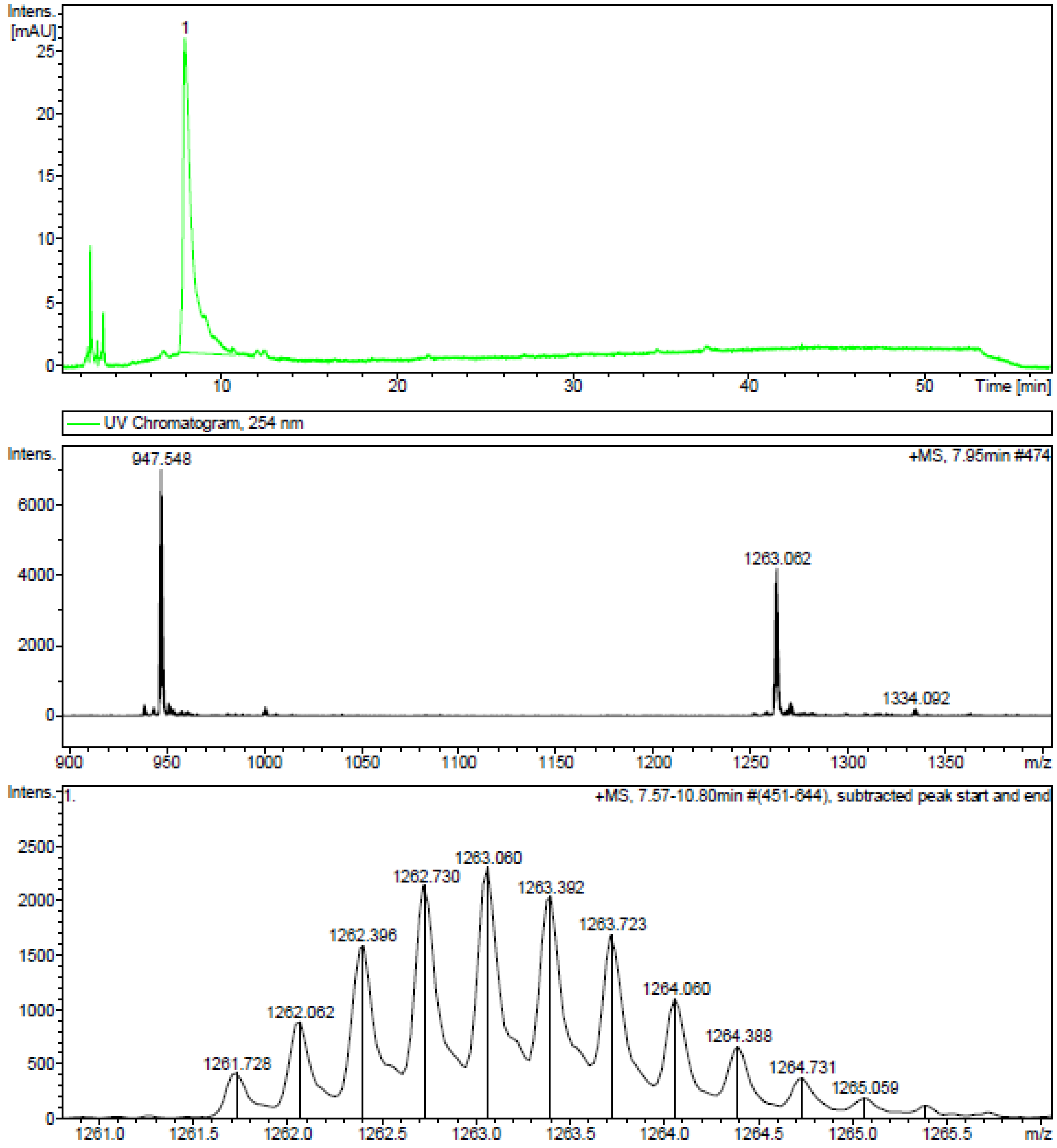

MS spectrum of compound **20**

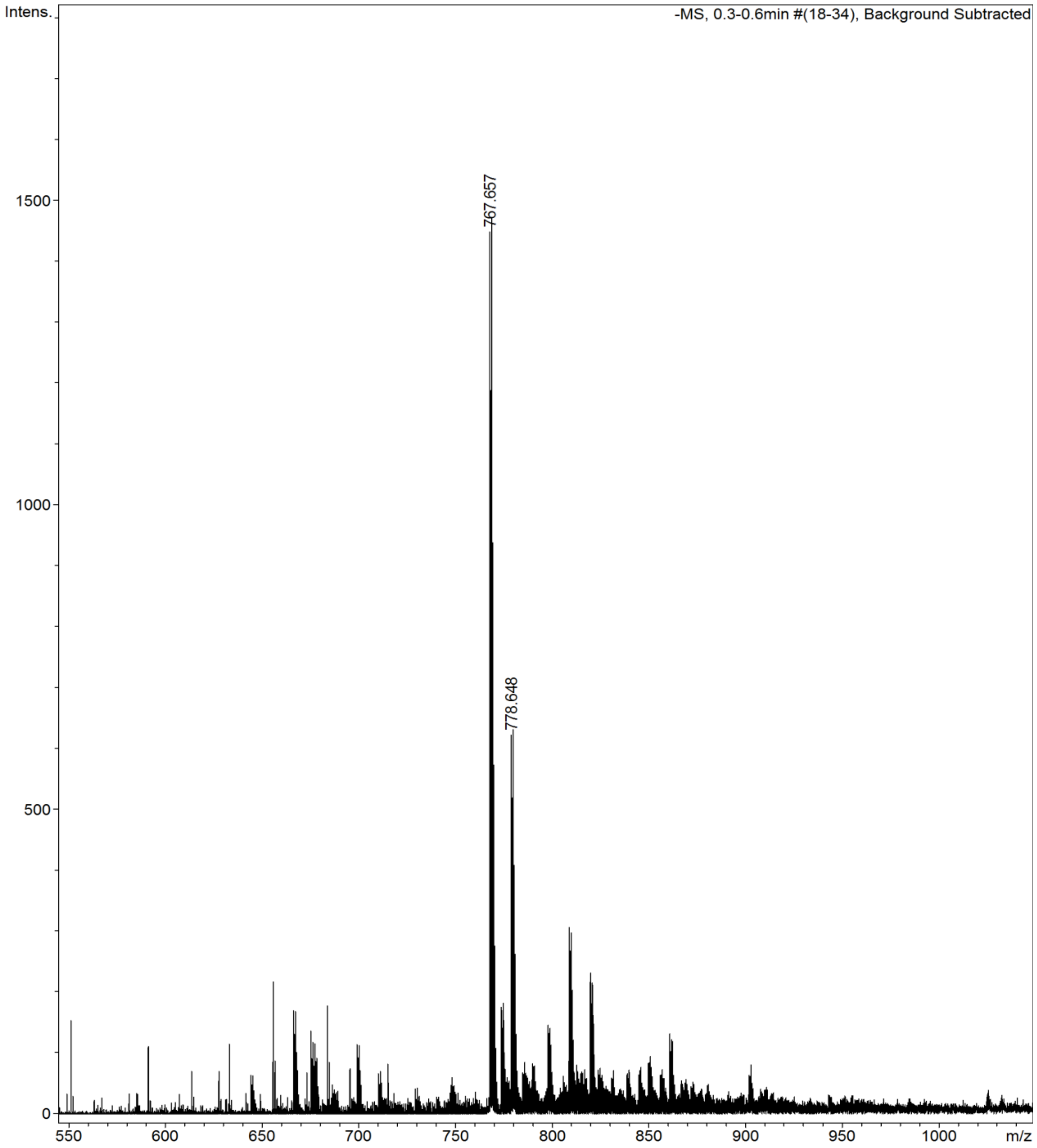

Supplement: Supplementary file 1 [file pharmaceuticals-15-00077-s001.zip › pharmaceuticals-1521801-supplementary.pdf]
